# Decision support systems for incurable non-small cell lung cancer: a systematic review

**DOI:** 10.1186/s12911-017-0542-1

**Published:** 2017-10-02

**Authors:** D. Révész, E. G. Engelhardt, J. J. Tamminga, F. M. N. H. Schramel, B. D. Onwuteaka-Philipsen, E. M. W. van de Garde, E. W. Steyerberg, E. P. Jansma, H. C. W. De Vet, V. M. H. Coupé

**Affiliations:** 1 Department of Epidemiology and Biostatistics, VU University Medical Center, PO Box 7057, 1007 MB Amsterdam, The Netherlands; 2 Department of Pulmonology, St Antonius Hospital, Nieuwegein, The Netherlands; 3 Department of Public and Occupational Health, and Palliative Care Expertise Centre, The EMGO Institute for Health and Care Research (EMGO+), VU University Medical Centre, De Boelelaan 1089a, 1081 HV Amsterdam, The Netherlands; 4 Department of Clinical Pharmacy, St. Antonius Hospital, Koekoekslaan 1, 3435 CM Nieuwegein, The Netherlands; 5 Department of Public Health, Centre for Medical Decision Making, Erasmus MC, Rotterdam, The Netherlands; 6 Medical Library, Vrije Universiteit, Amsterdam, The Netherlands; 7 Department of Epidemiology and Biostatistics, VU University Medical Center, EMGO Institute for Health and Care Research, De Boelelaan 1089a, 1081 HV Amsterdam, The Netherlands; 8 Department of Lung Diseases and Treatment, St. Antonius Hospital, Koekoekslaan 1, 3435 CM Nieuwegein, The Netherlands; 9 VU University Medical Center, Medical Information and Library, De Boelelaan 1117, 1081 HV Amsterdam, The Netherlands

**Keywords:** Decision support systems, Non-small-cell lung cancer, Survival, Prognosis

## Abstract

**Background:**

Individually tailored cancer treatment is essential to ensure optimal treatment and resource use. Treatments for incurable metastatic non-small cell lung cancer (NSCLC) are evolving rapidly, and decision support systems (DSS) for this patient population have been developed to balance benefits and harms for decision-making. The aim of this systematic review was to inventory DSS for stage IIIB/IV NSCLC patients.

**Methods:**

A systematic literature search was performed in Pubmed, Embase and the Cochrane Library. DSS were described extensively, including their predictors, model performances (i.e., discriminative ability and calibration), levels of validation and user friendliness.

**Results:**

The systematic search yielded 3531 articles. In total, 67 articles were included after additional reference tracking. The 39 identified DSS aim to predict overall survival and/or progression-free survival, but give no information about toxicity or cost-effectiveness. Various predictors were incorporated, such as performance status, serum and inflammatory markers, and patient and tumor characteristics. Some DSS were developed for the entire incurable NSCLC population, whereas others were specifically for patients with brain or spinal metastases. Few DSS had been validated externally using recent clinical data, and the discrimination and calibration were often poor.

**Conclusions:**

Many DSS have been developed for incurable NSCLC patients, but DSS are still lacking that are up-to-date with a good model performance, while covering the entire treatment spectrum. Future DSS should incorporate genetic and biological markers based on state-of-the-art evidence, and compare multiple treatment options to estimate survival, toxicity and cost-effectiveness.

**Electronic supplementary material:**

The online version of this article (10.1186/s12911-017-0542-1) contains supplementary material, which is available to authorized users.

## Background

According to the World Health Organization, cancer is one of the leading causes of morbidity and mortality worldwide, with lung cancer in the top five of cancers and the leading cause of cancer mortality with 1,6 million deaths in 2012 [1]. Roughly 80–85% of lung cancers are non-small cell lung cancer (NSCLC) [2]. Staging in patients is based on the Tumor Node Metastasis (TNM) classification, which is shown to be an important predictor of survival [3, 4]. Incurable patients with initial or recurrent metastatic NSCLC (stages IIIB and IV) have a short life expectancy, with 1-, 2- and 3-years survival ranging between 22 and 47%, 8–26% and 4–17%, respectively [5].

The current treatment of incurable NSCLC patients consists of systemic chemotherapy (CT), radiotherapy (RT), therapies targeting oncodrivers (e.g., epidermal growth factor receptor tyrosine kinase inhibitors, EGFR-TKI) or the immune system (immunotherapies), in addition to best supportive care (e.g., pain relief). In specific patient groups, mainly palliative surgery (for spinal metastases) and RT (for brain metastases) are advised. Palliative treatments aim to preserve or improve quality of life, lengthen life or decrease disease burden. Palliation can target the tumor tissue itself or symptoms, such as pain, diarrhea, obstipation, anxiety or depression. Individually tailored palliative cancer treatment is essential to ensure that patients receive the treatment that optimally matches their values and preferences, avoiding under- or overtreatment, and optimally utilizing available healthcare resources. However, this is a challenge due to the heterogeneity of the patient population, the multiple treatment options, and the marginal expected treatment benefits. Therefore, decision making in the palliative phase can be complex, as there is a delicate balance between benefits (e.g., symptom relief, life lengthening) and harms of treatments (e.g., side effects, loss of quality of life), as well as the costs of treatment. Decision support systems (DSS) could assist physicians in formulating an evidence-based treatment advice. DSS (e.g., prediction models, nomograms or decision trees) are based on statistical models in order to predict outcomes, such as overall survival (OS) (with our without treatment), toxicity and cost-effectiveness. They are based on patient and tumor characteristics, and preferably compare various treatment options. Research has shown that such clinical prediction models in end-of-life care are valued by physicians, because they enhance prognostic confidence and improve communication with patients, although they can also cause emotional distress in patients and raise prognostic overconfidence despite uncertainty in palliative care [6].

In patients with incurable NSCLC, some overviews have been published that summarize DSS in NSCLC patients. For instance, Mahar et al. have performed a systematic literature search from 1996 until 2015, identifying a total of 32 tools for all stages of lung cancer [7]. They described that the majority of the prediction models focus on NSCLC patients with metastatic disease, which can be explained by a larger need for DSS in this specific clinical population [7]. However, they did not use an extensive literature search with a large variety of MeSH headings, and thus, might have missed DSS for this subgroup. Other reviews have described DSS specifically developed either for patients with spinal metastases [8] or brain metastases, largely consisting of incurable NSCLC patients [9–12]. However, none of the earlier studies focused on the available DSS for the entire incurable NSCLC population, having short survival times due to rapidly progressive disease, whilst on the other hand there are rapid developments of new treatment options. Tools that aid clinical decision-making in this complex subgroup are urgently needed to help oncologists navigate the ever-growing maze of treatment options.

We conducted an extensive systematic literature search in order to summarize the available DSS for incurable patients with (initial or recurrent) metastatic NSCLC (stages IIIB and IV). We will give an overview of the development studies and the included predictors, as well as the levels of validation and calibration, and the model performances. Furthermore, we add concluding remarks about the user friendliness and ease of access of the identified DSS in clinical practice, and give direction to future research in this rapidly evolving field.

## Methods

### Literature search

A systematic literature search was performed in Pubmed, Embase and the Cochrane Library (until February 2016), in collaboration with the VU University Medical Center Medical Library. Titles and abstracts were retrieved and screened by three independent reviewers (DR, VC and JT), and discrepancies were resolved through consensus. After identification of potentially relevant papers, one researcher (DR) made the final selection of DSS by screening the full text papers, and when in doubt about inclusions VC, HdV, JT and FS were consulted. The search strategy consisted of a combination of database-specific MeSH terms, free text, ‘wild cards’ (words truncated by using “*”) and Boolean operators (“AND”, “OR”, “NOT”) (Additional file 1: Table S1). Inclusion and exclusion criteria are shown in Table 1. We followed the Preferred Reporting Items for Systematic Reviews and Meta-Analyses (PRISMA) Statement [13], which are listed in the Supplemental PRISMA checklist.

**Table 1:** Inclusion and exclusion criteria for literature search

Inclusion criteria for papers: - Describing development, validation or updating of a DSS; - Describing DSS that aims to predict prognosis (overall survival or progression-free survival), optimal treatment selection or toxicity; - Describing DSS that is depicted either as a risk score with a formula, nomogram, decision tree or (online) calculator or application. - Describing DSS that is applied in incurable patients with (initial or recurrent) metastatic NSCLC (stages IIIB and IV). - Describing DSS that is developed or validated in clinical data collected after 2000, as older data would not correctly reflect the current clinical practices anymore; - Papers published in English.
Exclusion criteria for papers: - Examining early stage NSCLC patients or patients with oligometastases; - Comparing treatments with curative intention or predicting whether curing metastases is still possible; - Describing univariate or multivariate analyses where only Hazard ratios or Odds ratios are reported, but no calculation of survival time for individual patients; - Non-original papers (e.g., reviews, methodological papers) only used for reference tracking; - Describing separate parameters that cannot be applied as an independent DSS; - Describing general tools that are not developed or validated in stage IIIB/IV NSCLC.

After selecting DSS from the systematic literature search, a manual search was performed to find the relevant development and validation studies for each DSS. First, references of the included papers were checked. Then, a ‘Cited by’ function and manual search were performed in Pubmed, using the following terms: “name DSS” AND “lung” AND “validate”. We also searched for additional DSS on a DSS indexing website (http://www.MedicalAlgorithms.com), and scanned the websites of the National Comprehensive Cancer Network (NCCN), European Society for Medical Oncology (ESMO), American Society of Clinical Oncology (ASCO), National Institute for Health and Care Excellence [14] and International Association for the Study of Lung Cancer (IASLC).

When many validation studies were found for a DSS, studies were selected when their sample size was >150 and/or they reported an impact analysis (i.e., prospectively treating patients according to a DSS, and evaluating the outcome).

### Data extraction

The quality of the included DSS was assessed by one researcher (DR) using an abbreviated CHARMS checklist [15], as shown in Table 2, and VC, HdV and JT were consulted for consensus when necessary. Model performance was assessed with measures for the discriminative ability of DSS (i.e., C-indices, area under the curve measures (AUC)), the Van Calster levels of calibration [16] and the Reilly levels of evidence [17]. Conclusions about user friendliness were drawn based on whether the predictors were routinely collected, whether individual scores could be easily calculated and whether additional online or paper tools are available.

**Table 2:** Quality assessment checklist for included DSS

Model performance
*Discriminative ability* Measuring how well DSS distinguishes between outcomes (e.g., risk groups) in external validations, using area under the ROC curve (AUC) analyses. AUC: <0.6 = poor; 0.6–0.7 = moderate; 0.7–0.8 = strong; >0.8 = very strong [18]
*van Calster levels of calibration (16)* Measuring how well predicted outcomes resemble observed outcomes: - Mean calibration – Correct average predicted risk. - Weak calibration – Correct average prediction effects. - Moderate calibration – Comparison between predicted and observed outcome. - Strong calibration – Event rate equals predicted risk for every covariate pattern.
*Reilly levels of evidence (17)* Measure for how thoroughly DSS is validated: - Level 1 – Derivation from a prediction model and not externally validated yet. - Level 2 – Narrow validation in one setting. - Level 3 – Broad validation in varied settings and populations. - Level 4 – Narrow impact analysis of model as decision rule in one setting. - Level 5 – Broad impact analysis of model as decision rule in varied settings and populations.
User friendliness
*Predictors routinely collected* Are all predictors in the DSS collected on a routine basis in clinical practice, or are special techniques needed?
*Easy use and access* Can the DSS easily be calculated (manually or using a computer) with an accessible regression formula, scoring system, nomogram, decision tree or online application?

## Results

### Overview retrieved articles

**Fig. 1:**
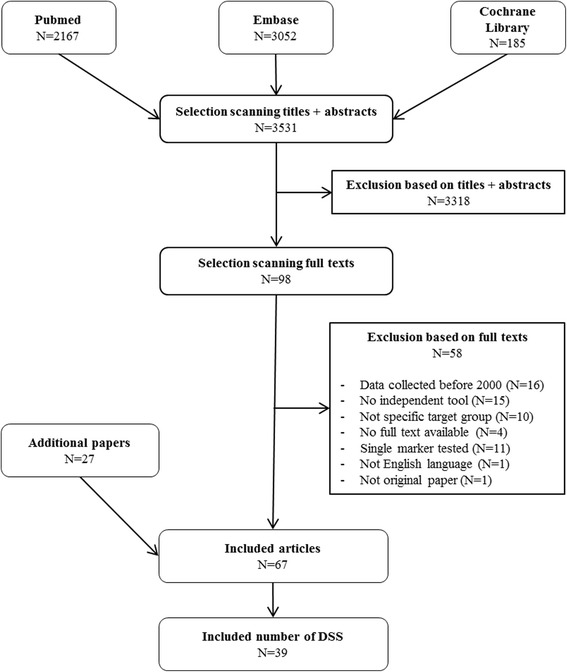
Flowchart of systematic literature search and article selection

With the systematic literature search a total of 3.531 articles were retrieved, of which 39 DSS were identified (Fig. 1). The included DSS were developed using data from a wide range of settings/contexts (i.e., clinical trials, prospective, and retrospective cohorts) and countries across most continents. Whereas some DSS can be used in all NSCLC patients (*N* = 21), others were specifically for patients with brain (*N* = 14), bone (N = 1) or spinal metastases (*N* = 3). Overall, these DSS aimed to predict prognosis (i.e., overall survival (OS) or progression-free survival (PFS)) in patients before or during treatments, such as systemic therapy (*N* = 6), RT, surgery and/or symptom management for brain metastases (N = 14) or for spinal metastases (N = 3), targeted therapies (N = 6), the general choice between tumor targeting vs. symptom management (N = 6), or mixed treatments (*N* = 4). No DSS were identified that predict the risk of side-effects or cost-effectiveness of treatments.

**Fig. 2:**
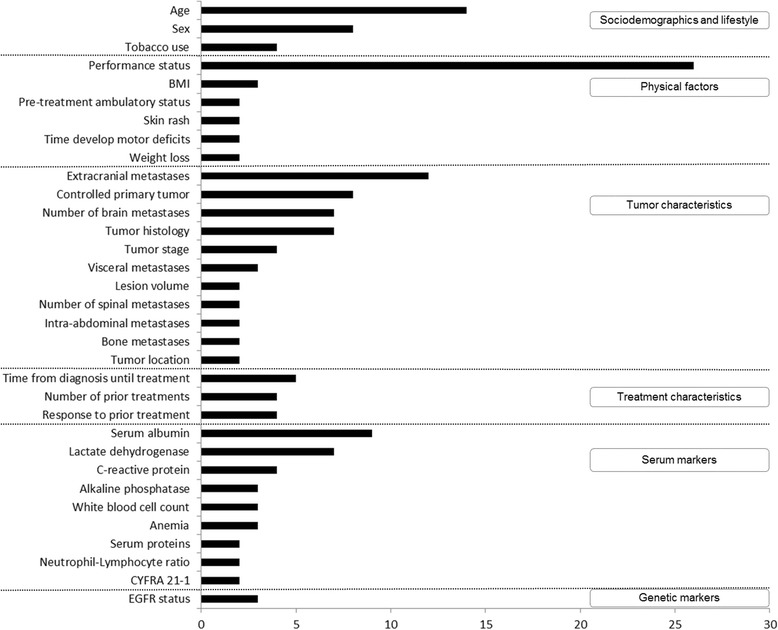
Frequency of predictors in DSS that were used at least two times classified in categories: sociodemographics and lifestyle, physical factors, tumor characteristics, treatment characteristics, serum and genetic markers. It must be noted that some predictors only apply to subgroups of patients with specific metastases or treatments

**Fig. 3:**
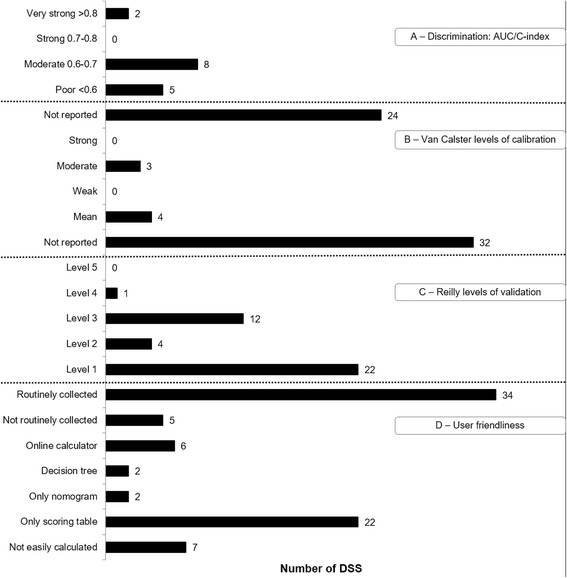
Frequency of DSS with discriminatory ability (A: area under the curve / C-index), levels of calibration (B), validation (C) and user friendliness (D: routine collection and ease of access)

Additional file 2: Table S2 gives an overview of the 39 included DSS for incurable NSCLC patients, alongside their development and validation studies, predictors and outcomes, model performances and user friendliness. The 39 DSS included a multitude of predictors, of which performance status, age, extracranial metastases and serum albumin levels were the most frequently incorporated in DSS, whereas *EGFR* status was the only genetic marker. Figure 2 shows the predictors that have been incorporated in at least two DSS. Next, Fig. 3 shows an overview of the variations in discriminatory ability (area under the ROC curve, AUC), the levels of validation and calibration in the retrieved DSS, and their user friendliness (i.e., routine collection and ease of access). The DSS that have been validated externally are discussed below and grouped based on the treatment options that they are developed for or validated in.

### DSS for general tumor targeting treatment vs. symptom management

A group of prognostic scores is based on the systemic inflammatory response, as chronic activation of this response typically goes along with negative outcomes (e.g., worse survival) [19]. Forrest et al. developed the Glasgow Prognostic Score (GPS, Table 3) that divides patients into three risk categories, with a higher score corresponding to shorter survival times [19]. Thereby, the GPS aids in deciding when it is worthwhile to provide intensive tumor-targeting treatment or merely symptom-management, although it is not clearly stated which risk category should not be treated intensively anymore [19]. Subsequently, various GPS-related models have been developed to measure the impact of inflammatory status on survival (scores are summarized in Table 3). The most frequently validated scores are the GPS, modified Glasgow Prognostic Score (mGPS) [20], and the Prognostic Index (PI) [21], but other scores that have not been validated externally were the advanced lung cancer inflammation index (ALI) [22], the Montreal Prognostic score [23, 24] and the Laboratory prognostic index (LPI) [25]. The GPS has an online calculator, which makes it easier to use in clinical practice [26]. Although the GPS predicts survival more accurately than the mGPS (higher AUC) [27], and its calibration is found to be better than the PI, its discriminative ability is found to be moderate with an AUC-value between 0.6–0.7 [28].

**Table 3:** DSS for choice between tumor targeting treatment vs. symptom management, based on inflammatory markers

	Alb	ALP	BMI	Ca	CRP	LDH	NLR	Stage	WBC	# groups
GPS^18^	√				√					3
mGPS^19^	√				√					3
PI^20^					√				√	3
ALI^21^	√		√				√			2
MPS^23^	√				√	√	√	√		3
LPI^24^	√	√		√		√			√	3

*Abbreviations*:*Alb* Albumin, *ALI* Advanced lung cancer inflammation index, *Alp* Alkaline phosphatase, *BMI* Body mass index, *Ca* Calcium, *CRP* C-reactive protein, *GPS* Glasgow prognostic score, *LDH* Lactate dehydrogenase, *LPI* Laboratory prognostic index, *mGPS* Modified GPS, *MPS* Montreal prognostic score, *NLR* Neutrophil/lymphocyte ratio, *PI* Prognostic index, *WBC* White blood cells

### DSS for systemic therapy

A few prognostic scores have been developed to select patients in which first line systemic therapy should be considered, such as the Hoang nomogram [29] and the Sanchez-Lara score [30], but these models have not been validated externally. Then, if patients manifest with disease progression after first line treatment, they might be suitable for second (or third) line treatment. For the latter group of patients, the Di Maio score was developed [23]. It takes various demographic and clinical factors into account, and divides patients into three prognostic groups (i.e., good, moderate and poor prognosis). However, the authors did not formulate any cut-offs for which groups should be considered for treatment. A 2012 validation study reported high discrimination (AUC = 0.926) for the Di Maio score [31]. However, the score is developed and validated with 10 year old clinical data, has not been validated in other settings, and there are no user friendly applications available.

### DSS for targeted therapy

The Florescu prognostic score was developed based on data from a clinical trial comparing erlotinib vs. placebo as a second or third line treatment [32]. Its estimates are based on patient, lifestyle, tumor, and treatment characteristics, and *EGFR* status. A total score can easily be calculated from the table in the paper, and it divides patients into four risk groups. The Florescu score was modified later in a small Polish sample [33], but only the original scoring system is validated in another patient group that received gefitinib, and none of these studies reported any measures of calibration or discrimination [34]. Moreover, the clinical data used in these studies was old (development study: 2001–2003 vs. validation study: 2003–2004), and no online applications are available. Nowadays, *EGFR* status would not be included into a model for EGFR-TKI treatment, as none of the patients without the mutation would be treated with EGFR-TKIs.

### DSS for RT, surgery and/or symptom management in patients with brain metastases

Other prognostic scores aim to predict prognosis in patients with brain metastases resulting from various primary tumor types, but the development and validation populations often included large numbers of NSCLC patients. The population with brain metastases is heterogeneous, and oncologists have to weigh harms and benefits for each individual patient in order to choose more intensive treatment or to stick to conservative symptom palliation. Various DSS have been developed to predict survival in incurable NSCLC patients with brain metastases who were undergoing whole brain radiotherapy (WBRT), stereotactic radiosurgery (SRS) or fractionated stereotactic radiotherapy (SRT) [10, 11, 35, 36], and are summarized in Table 4. First, the Recursive Partitioning Analyses (RPA) score was developed [37]. Later, the RPA score was modified twice into the modified RPA I and II [38, 39], and other DSS were derived, such as the Rotterdam score (RDAM) [40], Score Index for Radiosurgery (SIR) [41], Basic Score for Brain Metastases (BSBM) [42], modified BSBM score that included information regarding neurological complications and survival [43], Graded Prognostic Assessment (GPA) [44], Rades I [45] and Rades II score [46].

**Table 4:** DSS for RT, surgery and/or symptom management in patients with brain metastases

	Tumor type	KPS	ECM	Age	Sex	Tumor control	# lesions	Volume lesions	Time until RT	Response steroids	MD	NS	# groups
RPA^36^		√	√	√		√							3
RDAM^39^		√				√				√			3
SIR^40^		√		√		√	√	√					3
mRPA I^37^		√	√			√	√						5
BSBM^41^		√	√			√							4
GPA^43^		√	√	√			√						4
GGS^46^		√	√	√									4
Rades I^44^		√	√	√					√				4
ds-GPA^47^		√	√	√			√						4
Rades II^45^		√	√	√			√		√				3
BS nomogram^49^	√	√	√	√		√	√						–
mRPA II^38^		√	√			√	√						5
NSCLC-Rades^48^		√	√		√								3
mBSBM^42^		√	√			√	√	√			√	√	8

*Abbreviations:* BS Barnholtz-Sloan, *BSBM* Basic score for brain metastases, *ds-GPA* Disease-specific GPA, *ECM* Extracranial metastases, *GGS* Golden Grading System, *GPA* Graded prognostic assessment, *KPS* Karnofsky performance status, *mBSBM* Modified BSBM, *MD* Meningeal dissemination, *mRPA* Modified RPA, *NS* Neurological symptoms, *NSCLC* Non-small cell lung cancer, *RDAM* Rotterdam score, *RPA* Recursive partitioning analysis, *RT* Radiotherapy, *SIR* Score index for radiosurgery

After Golden et al. reported that primary tumor type is a significant predictor as well [47], they developed the Golden Grading System (GGS) in subsamples of patients with various primary tumors, including NSCLC [47]. Subsequently, various studies have been published with diagnosis-specific prognostic scores. The GPA has been tested in various populations (NSCLC, breast cancer and gastro-intestinal cancer), in order to create the disease-specific GPA (ds-GPA) [48], and Rades et al. developed the NSCLC-specific Rades score (NSCLC-Rades) [49]. A well-calibrated nomogram was created by Barnholtz-Sloan et al., incorporating primary tumor type into the model [50].

Some of the RPA-related scores have been validated repeatedly and tested for their discriminative ability in the literature. The reported AUC’s were moderate and varied between 0.5–0.7 [10, 11]. However, both the development papers and validation studies have often used relatively old clinical datasets (often before 2011), and the clinical relevance is therefore questionable. More recently, Lee et al. have suggested integrating *EGFR* mutational status into the ds-GPA score, which is a valuable step for future DSS [51]. Online tools are available for the RPA [52], GPA [53], and Barnholtz-Sloan nomogram [54]. Furthermore, the RPA score is incorporated in the ESMO guidelines and the Dutch national guidelines for brain metastases [55, 56].

### DSS for RT, surgery and/or symptom management in patients with spinal metastases

For patients with spinal metastases the Tokuhashi score was revised in 2005, and aims to support the oncologist when choosing between surgical interventions and a “more conservative approach” [57]. However, it was not precisely defined what a “more conservative approach” entailed [57]. This prognostic model divides patients into three prognostic groups, and gives an indication for treatment: excisional surgery for the lowest risk group, palliative surgery for the moderate risk group, and conservative treatment for the high risk group. The revised Tokuhashi score was externally validated in NSCLC patients [58, 59], had a mean level of calibration, but the authors did not report anything about the discrimination. A decision tree is provided [60], and the score can be found online as a calculator [61]. The revised Tokuhashi is incorporated in the Dutch national guidelines for spinal metastases [62], although the validation studies reported mixed results. An impact analysis of this score was performed by Tokuhashi et al., in which they followed up patients after they had received their treatment based on the scores’ prediction [58]. They found a large overlap of 87.9% between the predicted prognoses and the observed survival times [58]. On the other hand, in an external validation of Yu et al., the predicted and observed survival by the revised Tokuhashi score overlapped in merely 8.2% of the cases, indicating a poor predictive accuracy [59].

### DSS for mixed treatments

Few DSS have been developed in patient populations with mixed treatment options, but have not been externally validated, such as the Daniele score [63], Lin score [64] and Zhang score [65]. Only the Blanchon model [66] has been validated in another population [67], thereby, reaching Reilly level 2. This scoring is developed to predict risk of death at four years in patients with various treatments (i.e., CT, surgery, RT, combinations). However, the calibration of this model has not been reported, and its discriminatory ability was moderate (AUC = 0.61) in the external validation study [67]. Furthermore, no online tools are available for this DSS.

## Discussion

Decision support systems (DSS) aid clinical decision-making by comparing various treatment options, and by predicting harms and benefits based on patient and tumor characteristics. This systematic review provides a comprehensive overview of DSS developed and/or validated for incurable patients with (initial or recurrent) metastatic NSCLC (stages IIIB and IV). In total, 39 DSS have been identified, of which 17 had been externally validated. Each DSS is described, and an overview is given of their discrimination, calibration and user friendliness. These DSS estimate OS and/or PFS, and are based on patient and treatment characteristics, sociodemographic, lifestyle and physical factors, serum markers, and to a lesser extent genetic markers. Regardless of the relatively large amount of existing DSS, there is room for improvement in the tools for clinical decision-making.

Less than half of the currently available DSS have been externally validated in a broader setting, and most validations have also been performed in relatively old datasets. Validated tools also showed poor model performances. Another shortcoming of the currently available DSS is that they only estimate OS or PFS, but do not incorporate other outcomes of societal relevance, such as toxicity or cost-effectiveness. In line with previous reviews, none of the DSS weigh the risks and benefits of treatments [7, 9–12]. This makes decision-making difficult, as not all facets are discussed. It should be kept in mind that DSS only aim to facilitate the decision-making process. Physician can use information obtained from DSS to derive a treatment advice, or to inform patients during consultations. The information obtained from DSS can help patients develop informed preferences, which are the basis for shared decision-making. It is therefore important for DSS to at least provide information of both the benefits (in terms of survival) and harms (in terms of side-effects) of treatment.

Also, most tools are developed to give a rough estimate of survival, either in the entire incurable NSCLC population, for systemic therapy, targeted therapy, mixed treatments or specifically for patients with brain or spinal metastases. There is no DSS that gives an overview of all treatments relevant to consider in the incurable NSCLC population (or a specific subgroup), or that offers clear cut-off points for when it is worthwhile to provide intensive treatment or best supportive care. In the meantime, studies often lack good definition of the control conditions, which are described as a ‘more conservative approach’ or ‘best supportive care’. Even though clinical guidelines also describe all available treatment options, they do not present overviews that enable individualized decision making. Some currently identified DSS are incorporated in existing guidelines, although these tools’ performance is mediocre. For instance, the RPA was extensively examined in multiple studies and is incorporated in the ESMO guidelines [56] and the Dutch Oncoline guidelines for brain metastases [55], but its discriminative ability is not at all strong. The revised Tokuhashi score is only mentioned in the Dutch national guidelines for spinal metastases [62], although the accuracy of this DSS is not consistently good in all studies [58, 59]. For more personalized clinical decision-making, guidelines would ideally incorporate available DSS based on recent clinical evidence with good discriminatory ability and calibration that compare multiple treatment options, and present multiple outcomes (e.g., benefits, harms and cost-effectiveness).

Within this review, we found that DSS that outperformed others (e.g., Di Maio score with AUC = 0.926) or that have a user friendly lay-out (e.g., Barnholtz-Sloan nomogram) are not validated in broader settings, and have not been tested extensively. An explanation for the lack of optimal tools could be that the current process of development, validation and updating of DSS is too time-consuming. Therefore, DSS are expected to be outdated by the time that extensive validations can be performed due to the rapid developments in lung cancer care. Other methods, such as rapid learning techniques [68, 69] and other sophisticated algorithms might be developed for more continuous updating and validating procedures. Another explanation for the relatively poor model performances might also be that survival in this heterogeneous group of patients cannot be estimated with high accuracy. Even though they aim to personalize decision-making, DSS are based on statistical models that by definition make use of probabilities. These models generally describe the association between an outcome and a very limited set of potential predictors only. By adding a broader range of predictors from large longitudinal databases to build and validate models, perhaps the biological complexity and heterogeneity can be better reflected in the resulting outcome predictions. The use of biomarkers might lead to higher accuracy than the more general predictors such as age and tobacco use.

In the last decennia, the concept of personalized medicine has taken a more central position in metastasized cancer care. Therefore, future DSS should take into account specific biological markers and genes, such as *EGFR* and *ALK*. The complexity of gene mutations, translocations and rearrangements can explain why some treatments are effective, while others induce little response in patients. Some systematic reviews have summarized the currently known and relevant NSCLC genetic markers (e.g., *EGFR, EML4/ALK mutations*), and other markers for which there is insufficient evidence for use in clinical decision-making (e.g., *K-RAS*, *ERCC1* *, BRCA, Beta tubulin III, RRM1, TP-53* mutations) [70–72]. Genetic markers will become increasingly important in the future in order to distinguish between responders and non-responders. The same is true for immunotherapeutic approaches, as for example, the NCCN guidelines recommend immune checkpoint inhibitors for incurable NSCLC patients, based on performance status and treatment responses [73]. These guidelines give some insights and flow diagrams about the application of two new immunotherapeutic agents, nivolumab and pembrolizumab that target the programmed cell death protein 1 (PD-1) pathway. However, important insights are still lacking to determine for which patients immunological treatments are (most) effective, especially considering that the targeted treatments are costly and some induce severe side effects.

The current systematic review aimed to shed light on which tools are available, and which gaps remain to be filled in future research. In general, the available DSS are of limited value to daily clinical practice because they used relatively old clinical data (before 2000), focused more on advantages than disadvantages of merely one or two treatment options, and still lack available user friendly applications. By collaborating with national databases, a continuous updating procedure could be incorporated as well. Recently some research groups have made new web-based prediction tools that shed light on both the advantages and disadvantages of treatments. One example is the Predict Cancer website [74], where an application is presented including DSS for lung, rectum, head and neck cancer and brain metastases. This application aims to support oncologists with the estimation of expected survival rates, side effects of treatments, cost-effectiveness of a treatment plan, and other important parameters. As another example, the ASCO, ESMO, NCCN and Institute for Clinical and Economic Review (ICER) have presented frameworks, in which not only benefits but also the cost-effectiveness and toxicity of several anti-cancer drugs were quantified [75, 76]. Furthermore, Warner et al. have created a novel and promising rapid learning system for various cancer types (including lung cancer) that automatically calculates and displays mutation-specific survival rates from electronic health record data (69). These approaches could be useful for future DSS too.

Strengths of the current systematic review are the extensive literature search performed both in online databases and with reference tracking. We have chosen a specific population in which decision support can be of great value because of the short survival time, rapid treatment-related developments, and complex decisions about when it still is in the patients’ best interest to provide invasive treatment, or when the transition needs to be made to provide only symptom relief. Nevertheless, some limitations have to be taken into account as well. Terminology to describe DSS varies greatly throughout the field, and this could have hampered the ability to find all existing DSS even with an extensive search strategy. Also, we have not used the complete CHARMS checklist [15] to assess methodological quality of the included DSS, as many items were not reported. Instead we created an abbreviated list of items that covers the main aspects of model performance (shown in Table 4).

In the current study, 39 DSS have been identified for incurable metastatic NSCLC patients. Previously, Mahar et al. have performed a systematic literature search, and found 32 tools both for small cell lung cancer (*N* = 7) and NSCLC (*N* = 25) [7]. Of the tools developed for NSCLC patients, there was one tool for all tumor stages, eight tools for stages I-III, and 16 for advanced/incurable disease [7]. Mahar et al. found 16 tools for our target population, of which three were excluded in our current search: two were based on older clinical data (before 2000), and one only reported hazard ratios and no formulas for individual probabilities. Furthermore, three of their included tools were published together in one paper, and were summarized as one tool in our current study [77]. To conclude, our systematic literature search identified 28 additional studies, and added extensive information about the studies that have externally validated these DSS, the user friendliness and the application methods of the tool in clinical practice.

## Conclusions

Our study adds to the current knowledge in the field of DSS in incurable NSCLC, as conclusions are drawn about the extent and quality of validation (i.e., Reilly levels of validation), extent of calibration (i.e., Van Calster levels of calibration) and user friendliness (i.e., routine collection, ease of use, online tools). In addition, an overview is given of the used predictors, grouped into domains. Not only does our review increases knowledge of existing DSS, but we also indicate areas for improvement. Overall, we can conclude that multiple DSS have been developed for incurable patients with (initial or recurrent) metastatic NSCLC (stages IIIB and IV), but most of them have used relatively old clinical data, focused on benefits rather than harms in terms of toxicity and risks, did not compare various treatment options, control treatments (conservative treatment, best supportive care, usual care) were often poorly or not described, have not been (externally) validated or still lack available user friendly applications (i.e., scoring tables, online calculators, mobile applications). Also, various predictors in the domains of serum markers, tumor and treatment characteristics have been included, but apart from *EGFR* in one DSS, biological markers for targeted and immunotherapies are still lacking. Other methods might be available for future DSS designs in order to incorporate all relevant individual characteristics efficiently, while taking into account the needs of oncologists in their daily practice. Preferably, future DSS provide oncologists an efficient method to stay up-to-date with the rapid innovations in lung cancer care.

## Additional files

Additional file 1: Table S1. Detailed search strategy per database in order to find all published decision support systems for incurable patients with (initial or recurrent) metastatic non-small cell lung cancer. (DOCX 15 kb)

Additional file 2: Table S2. Overview and quality assessment of decision support systems for incurable patients with (initial or recurrent) metastatic non-small cell lung cancer. (DOCX 346 kb)

## Abbreviations

ALI: Advanced lung cancer inflammation index
Alp: Alkaline phosphatase
AUC: Area Under the Curve
BMI: Body mass index
BS: Barnholtz-Sloan
BSBM: Basic Score for Brain Metastases
CA: Cancer antigen
CEA: Carcinoembryonic antigen
C-index: Concordance index
CRP: C-reactive protein
CT: Chemotherapy
CYFRA 21-1: Cytokeratin-19 fragments
ds-GPA: Disease specific Graded Prognostic Assessment
DSS: Decision Support System(s)
ECM: Extracranial metastases
EGFR: Epidermal growth factor receptor
EGFR-TKI: Epidermal growth factor receptor tyrosine kinase inhibitors
GGS: Golden Grading System
GPA: Graded Prognostic Assessment
GPS: Glasgow prognostic score
HrQL: Health-related quality of life
KPS: Karnofsky Performance status
LDH: Lactate dehydrogenase
LPI: Laboratory prognostic index
mGPS: Modified GPS
M: Months
MPS: Montreal prognostic score
NLR: Neutrophil/Lymphocyte ratio
NSCLC: Non-small cell lung cancer
NSCLC-Rades: NSCLC-specific Rades
OS: Overall survival
PFS: Progression-free survival
PI: Prognostic index
PS: Performance status
RDAM: Rotterdam prognostic score
RPA: Recursive Partitioning Analysis
RT: Radiotherapy
RTOG: Radiation Therapy Oncology Group
SIR: Score Index For Radiosurgery
SRS: Stereotactic radiosurgery
SRT: (Fractionated) Stereotactic radiotherapy
TKI: Tyrosine kinase inhibitors
TNM: Tumor, Node, Metastasis classification
WBRT: Whole brain radiotherapy
