# Supplementary material for: Decision support systems for incurable non-small cell lung cancer: a systematic review
Source: BMC Med Inform Decis Mak. 2017 Oct 2;17:144. doi: 10.1186/s12911-017-0542-1 (PMC5625762; doi:10.1186/s12911-017-0542-1)
Supplement: Supplementary file 1 — Detailed search strategy per database in order to find all published decision support systems for incurable patients with (initial or recurrent) metastatic non-small cell lung cancer. (DOCX 15 kb) [file 12911_2017_542_MOESM1_ESM.docx]

| **Supplement Table 1: Detailed search strategy per database in order to find all published decision support systems for incurable patients with recurrent metastatic Non-Small-Cell Lung Carcinoma** |
| --- |
| ***Pubmed***  *"Decision Support Systems, Clinical"[Mesh] OR "Decision Support Techniques"[Mesh] OR "Nomograms"[Mesh] OR "Markov Chains"[Mesh] OR Decision Support System*[tiab] OR Decision Support Technique*[tiab] OR Decision Aid*[tiab] OR Decision Support Model*[tiab] OR Decision Analys*[tiab] OR Decision Modeling[tiab] OR nomogram*[tiab] OR Prediction Rule*[tiab] OR (prognos*[tiab] AND (index[tiab] OR score*[tiab] OR model*[tiab])) OR markov[tiab] AND ("Prognosis"[Mesh:noexp] OR "incidence"[MeSH Terms:noexp] OR mortality[MeSH Terms] OR follow up studies[MeSH:noexp] OR prognos*[tiab] OR predict*[tiab] OR course*[tiab] OR mortalit*[tiab]) OR "Life Expectancy"[Mesh] OR "Survival Rate"[Mesh] OR "Longevity"[Mesh] OR "Longevity"[tiab] OR "Life Expectancy"[tiab] OR "life expectance"[tiab] OR "life expectation"[tiab] OR "Survival"[tiab] OR prognos*[tiab] OR (toxicit*[tiab] OR toxic potential*[tiab] OR "margin of safety"[tiab] OR adverse effect*[tiab] OR survival[tiab]) AND "Carcinoma, Non-Small-Cell Lung"[Mesh] OR Non-Small-Cell Lung[tiab] OR NSCLC[tiab]* |
| ***Embase***  *'decision support system'/exp OR 'nomogram'/exp OR 'probability'/exp OR 'decision support system*':ab,ti OR 'decision support technique*':ab,ti OR 'decision aid*':ab,ti OR 'decision support model*':ab,ti OR 'decision analys*':ab,ti OR decision AND modeling:ab,ti OR 'prediction rule*':ab,ti OR probabilit*:ab,ti OR nomogram*:ab,ti OR (prognos*:ab,ti AND (index:ab,ti OR score*:ab,ti OR model*:ab,ti)) OR markov:ab,ti AND 'prognosis'/exp OR 'incidence'/exp OR 'mortality'/exp OR 'follow up'/exp OR predict*:ab,ti OR course*:ab,ti OR mortalit*:ab,ti OR 'life expectancy'/exp OR 'survival rate'/exp OR 'longevity'/exp OR 'longevity':ab,ti OR 'life expectancy':ab,ti OR 'life expectance':ab,ti OR 'life expectation':ab,ti OR 'survival':ab,ti OR prognos*:ab,ti OR toxicit*:ab,ti OR 'toxic potential*':ab,ti OR 'margin of safety':ab,ti OR 'adverse effect*':ab,ti AND 'non small cell lung cancer'/exp OR 'non-small-cell lung':ab,ti OR nsclc:ab,ti AND ('article'/it OR 'article in press'/it OR 'review'/it)* |
| ***Cochrane library***  *"Non-Small-Cell Lung" or NSCLC:ti,ab,kw AND "incidence" or mortality or follow up studies or prognos* or predict* or course* or mortalit* or "Life Expectancy" or "Longevity" or "Life Expectancy" or "life expectance" or "life expectation" or "Survival" or toxicit* or toxic potential* or "margin of safety" or adverse effect* or survival:ti,ab,kw AND "Decision Support System*" or "Decision Support Technique*" or "Decision Aid*" or "Decision Support Model*" or "Decision Analys*" or "Decision Modeling OR Prediction Rule*" or nomogram* or (prognos* and (index or score* or model*)) or markov:ti,ab,kw* |
