# Supplementary material for: Decision support systems for incurable non-small cell lung cancer: a systematic review
Source: BMC Med Inform Decis Mak. 2017 Oct 2;17:144. doi: 10.1186/s12911-017-0542-1 (PMC5625762; doi:10.1186/s12911-017-0542-1)
Supplement: Supplementary file 2 — Overview and quality assessment of decision support systems for incurable patients with (initial or recurrent) metastatic non-small cell lung cancer. (DOCX 346 kb) [file 12911_2017_542_MOESM2_ESM.docx]

| Supplement Table 2: Overview and quality assessment of decision support systems for incurable patients with recurrent metastatic non-small cell lung cancer | | | | | | | |
| --- | --- | --- | --- | --- | --- | --- | --- |
| **Name DSS** | **Development** | **Aim** | **Predictors** | **Output** | **Validation** | **Model performance** | **User friendiness** |
| Adverse prognostic factors (APF)(1) | Prospective: 1999-2004 (Spain)  N=135 NSCLC (N=38 stage IIIB, N=87 stage IV) patients with/without CT | Predict OS, for systemic therapy | - CT  - CA125  - CYFRA 21-1  - ECOG PS  - Leukocytes  - Metastases | 3 prognostic groups:  - Good: 0-1 🡪 15 m  - Moderate: 2-3 🡪 6 m  - Poor: >3 🡪 2 m | - | Discriminative ability: -  Van Calster level of calibration: NR  Reilly level of evidence: Level 1 | - Not routinely collected  - Manual calculation not easy  - No online tool |
| Armero Disability model(2) | Prospective: 2008-2010 (Spain)  N=35 stage IV NSCLC patients receiving conventional CT | Predict OS and PFS from diagnosis, for systemic therapy | - Age  - Anemia  - BMI  - CEA  - Histology  - LDH  - Number affected organs  - Sex  - Tumor location | 3 transition groups:  1) Stage IV  2) Progression  3) Death | *-* | Discriminative ability: -  Van Calster level of calibration: NR  Reilly level of evidence: Level 1 | - Routinely collected  - Manual calculation not easy  - No online tool |
| Blanchon prognostic index(3) | Prospective: 2000-2005 (France)  N=4479 NSCLC (N=1466 stages IIIA/IIIB; N=1799 stage IV), divided into development cohort (N=2979) and validation cohort (N=1500), mixed treatments (CT, palliative, surgery, RT, combinations) | Predict risk of death at 4 years, for mixed treatments | - Age  - ECOG PS  - Histology  - Sex  - Stage | 6 risk groups:  1) Lowest: 0-1 🡪 35-36% risk  2) 2-4 🡪 59-60%  3) 5-7 🡪 77%  4) 8-10 🡪 88-89%  5) 11-14 🡪 96-97%  6) Highest: >14 🡪 99% | Clinical trials: 1988-2009 (US)(4)  N=3671 NSCLC patients (N=1611 stage IV) in phase II/III clinical trials for mixed treatments (CT, RT, surgery) | Discriminative ability:  Internal(3)  Develop AUC=0.85  Validation AUC=0.86  External(4)  AUC=0.61  Van Calster level of calibration: NR  Reilly level of evidence: Level 2 | - Routinely collected  - Manual calculation not easy  - No online tool |
| Daniele score(5) | Retrospective: 1999-2012 (Italy)  N=661 deceased patients with bone metastases, after mixed treatments (1^st^ line CT, EGFR-TKI) | Predict OS, for mixed treatments | - Age  - ECOG PS  - Non-adenocarcinoma  histology  - Visceral metastases | 2 prognostic groups:  - Good: 0-2 factors 🡪 8 m  - Poor: >2 factors 🡪 5 m | - | Discriminative ability: -  Van Calster level of calibration: NR  Reilly level of evidence: Level 1 | - Routinely collected  - Easy manual calculation  - No online tool |
| **Footnotes**: BMI= Body mass index; CA= Cancer antigen; CEA= Carcinoembryonic antigen; CT= Chemotherapy; CYFRA 21-2=Cytokeratin-19 fragments; EGFR-TKI= Epidermal growth factor receptor tyrosine kinase inhibitors; LDH= Lactate dehydrogenase; M= Months; NR= Not reported; NSCLC= Non-small cell lung cancer; OS= Overall survival; PFS= Progression-free survival; PS= Performance status; RT= Radiotherapy | | | | | | | |

| Continued Supplement Table 2: Overview and quality assessment of decision support systems for incurable patients with recurrent metastatic non-small cell lung cancer | | | | | | | |
| --- | --- | --- | --- | --- | --- | --- | --- |
| **Name DSS** | **Development** | **Aim** | **Predictors** | **Output** | **Validation** | **Model performance** | **User friendiness** |
| Di Maio score(6) | RCT: 1999-2007 (Italy, Netherlands, Greece, France, Taiwan, Japan)  N=1197 stage IIIB/IV NSCLC patients before 2^nd^ line CT | Predict OS, for systemic therapy | - Age  - Histology  - Platinum-  based 1^st^ line CT  - Response to prior CT  - Sex  - Stage | 3 prognostic groups:  - Best (0-4) 🡪 11.6 m  - Middle (5-9) 🡪 7.5 m  - Worst (>9) 🡪 3.0 m | RCT: 2003-2005 (Italy)(7)  N=551 stage IIIB/IV NSCLC before vinflunine vs. docetaxel as 2^nd^ line CT | Discriminative ability:  Internal(6)  AUC=0.643  External(7)  AUC=0.926  Van Calster level of calibration: NR  Reilly level of evidence: Level 2 | - Routinely collected  - Easy manual calculation  - No online tool |
| Florescu score(8) | RCT: 2001-2003 (Canada)  N=731 stage IIIB/IV NSCLC receiving erlotinib vs. placebo | Predict OS, for targeted therapy | - Anemia  - ECOG PS  - EGFR status  - Ethnicity (oriental vs. other)  - LDH  - Number of prior treatments  - Response to prior CT  - Smoking  - Time interval from diagnosis  - Weight loss | 4 risk groups:  - Low (<18) 🡪 20.6 m  - Mid-low (18-27) 🡪 10.4 m  - Mid-high (28-38) 🡪 4.1 m  - High (>38) 🡪 1.9 m | Retrospective: 2003-2004 (China)(9)  N=119 locally advanced or metastatic NSCLC receiving gefitinib | Discriminative ability:  External(9)  Van Calster level of calibration: NR  Reilly level of evidence: Level 2 | - Routinely collected  - Easy manual calculation  - No online tool |
| Modified Florescu score(10) | Prospective: 2007-2010 (Poland)  N=73 stage IIIB/IV NSCLC receiving erlotinib | Predict OS, for targeted therapy | - Anemia  - ECOG PS  - EGFR status  - LDH  - Number of prior treatments  - Sex  - Skin rash  - Smoking  - Time interval from diagnosis  - Weight loss | 4 risk groups:  - Low (<16) 🡪 19 m  - Mid-low (17-32) 🡪 9 m  - Mid-high (33-44) 🡪 3.3 m  - High (>44) 🡪 1.5 m | - | Discriminative ability: -  Van Calster level of calibration: NR  Reilly level of evidence: Level 1 | - Routinely collected  - Easy manual calculation  - No online tool |
| **Footnotes**: BMI= Body mass index; CA= Cancer antigen; CEA= Carcinoembryonic antigen; CT= Chemotherapy; LDH= Lactate dehydrogenase; M= Months; NR= Not reported; NSCLC= Non-small cell lung cancer; OS= Overall survival; PS= Performance status; RCT= Randomized clinical trial; | | | | | | | |

| Continued Supplement Table 2: Overview and quality assessment of decision support systems for incurable patients with recurrent metastatic non-small cell lung cancer | | | | | | | |
| --- | --- | --- | --- | --- | --- | --- | --- |
| **Name DSS** | **Development** | **Aim** | **Predictors** | **Output** | **Validation** | **Model performance** | **User friendiness** |
| Systemic inflammatory response scores: A t/m F  A) Glasgow Prognostic Score (GPS)(11) | Retrospective + prospective: 1997-2002 (England)  N=161 stage III/IV NSCLC receiving active treatment (cisplatin-based CT and/or radical RT) vs. palliative treatment (RT and/or symptom control) | Predict OS and PFS (1-5 years), for tumor-targeting treatment vs. symptom management | - Albumin  - CRP | 3 groups based on number of risk factors  0 🡪 17.0 m  1 🡪 8.9 m  2 🡪 3.9 m | Prospective: 2008-2011 (China)(12)  Retrospective: 2011-2015 (China)(13)  N=138 stage IIIB/IV NSCLC patients before CT(12)  N=2988 NSCLC (N=1745 inoperable patients: N=471 stage III, N=1074 stage IV) receiving CT and/or RT(13) | Discriminative ability:  AUC=0.713 (13)  PFS AUC=0.62 (12)  OS AUC=0.66 (12)  Van Calster level of calibration: Moderate  Reilly level of evidence: Level 3 | - Routinely collected  - Easy manual calculation  - Online calculator tool(14) |
| B) Modified Glasgow Prognostic Score (mGPS)(15) | Retrospective: 1997-2004 (England)  N=316 CRC after surgery | Predict OS and PFS (1-5 years), for tumor-targeting treatment vs. symptom management | - Albumin  - CRP | 3 groups based on number of risk factors  *Score 1 only with elevated CRP*  0) inoperable: 20 m  1) inoperable: 10 m  2) inoperable: 3 m (13) | Retrospective: 2011-2015 (China)(13)  N=2988 NSCLC (N=1745 inoperable patients: N=471 stage III, N=1074 stage IV) receiving CT and/or RT | Discriminative ability:  External(13)  AUC=0.690  Van Calster level of calibration: NR  Reilly level of evidence: Level 3 | - Routinely collected  - Easy manual calculation  - No online tool |
| C) Prognostic index (PI)(16) | Prospective: 2005-2009 (Canada)  N=303 stage IV NSCLC receiving 2 cycles platinum-doublet CT | Predict OS and PFS (1-5 years), for tumor-targeting treatment vs. symptom management | - CRP  - White blood cells | 3 groups based on number of risk factors  0) 20.0 m  1) 10.4 m  2) 7.9 m | Prospective: 2008-2011 (China)(12)  N=138 stage IIIB/IV NSCLC patients before CT | Discriminative ability:  External(12)  PFS AUC=0.57  OS AUC=0.56  Van Calster level of calibration: Moderate  Reilly level of evidence: Level 3 | - Routinely collected  - Easy manual calculation  - No online tool |
| D) Advanced lung cancer inflammation index (ALI)(17) | Retrospective: 2000-2011 (US)  N=173 stage IV NSCLC receiving CT | Predict OS and PFS (1-5 years), for tumor-targeting treatment vs. symptom management | - Albumin  - BMI  - NLR | 2 inflammation groups:  BMI x Albumin / NLR  OS  ≥ 18: low 🡪 8.3 m  < 18: high 🡪 3.4 m  PFS  ≥ 18: low 🡪 5.1 m  < 18: high 🡪 2.4 m | - | Discriminative ability: -  Van Calster level of calibration: NR  Reilly level of evidence: Level 1 | - Routinely collected  - Easy manual calculation  - No online tool |
| **Footnotes**: AUC= Area under the ROC curve; BMI= Body mass index; CRP= C-reactive protein; CT= Chemotherapy; LDH= Lactate dehydrogenase; M= Months; NLR= Neutrophil/Lymphocyte ratio; NR= Not reported; NSCLC= Non-small cell lung cancer; OS= Overall survival; PFS= Progression-free survival; PS= Performance status; RCT= Randomized clinical trial; RT= Radiotherapy | | | | | | | |

| Continued Supplement Table 2: Overview and quality assessment of decision support systems for incurable patients with recurrent metastatic non-small cell lung cancer | | | | | | | |
| --- | --- | --- | --- | --- | --- | --- | --- |
| **Name DSS** | **Development** | **Aim** | **Predictors** | **Output** | **Validation** | **Model performance** | **User friendiness** |
| E) Montreal prognostic score (18) | Retrospective: 2002-2013 (Canada)  N=258 (test cohort, 2002-2008) and N=433 (validation cohort, 2006-2013) stage III/IV NSCLC patients before CT | Predict OS and PFS (1-5 years), for tumor-targeting treatment vs. symptom management | - Albumin  - CRP  - LDH  - NLR  - Stage | 3 risk groups:  Testing cohort  1) Low: 0-3 🡪 18.2 m  2) Middle: 4-12 🡪 8.2 m  3) High: 13 🡪 2.5 m  Validation cohort  1) Low: 0-3 🡪 22.3 m  2) Middle: 4-12 🡪 7.5 m  3) High: 13 🡪 3.3 m | - | Discriminative ability: -  Van Calster level of calibration: NR  Reilly level of evidence: Level 1 | - Routinely collected  - Easy manual calculation  - No online tool |
| F) Laboratory prognostic index (LPI)(19) | Retrospective: 2000-2010 (Turkey)  N=462 stage IIIB/IV NSCLC patients receiving CT, RT or best supportive care | Predict OS and PFS (1-5 years), for tumor-targeting treatment vs. symptom management | - Albumin  - ALP  - Calcium  - LDH  - White blood count | 3 groups based on number of risk factors  OS  0) 19 m  1) 11 m  ≥2) 7 m  PFS  0) 10 m  1) 7 m  ≥2) 5 m | - | Discriminative ability: -  Van Calster level of calibration: NR  Reilly level of evidence: Level 1 | - Routinely collected  - Easy manual calculation  - No online tool |
| Hoang nomogram(20) | Clinical trial: 2001-2004 (US)  N=850 stage IIIB/IV NSCLC before 1^st^ line paclitaxel and carboplatin with /without bevacizumab | Predict OS and PFS (6 m – 1 year), for systemic therapy | Nomogram OS  - Adrenal metastases  - Albumin  - Bevacizumab  - BMI  - Bone / bone marrow metastases  - LDH  - Mediastinal metastases  - Non-adeno/bronchoalveolar carcinoma  - PS  - Sex  - Skin metastases  Nomogram PFS  - Albumin  - Bevacizumab  - Bone (marrow) metastases  - Liver metastases  - Mediastinal metastases  - PS  - Skin metastases | Percentage 1-year OS and 6-m PFS | - | Discriminative ability: -  Van Calster level of calibration: Mean  Reilly level of evidence: Level 1 | - Routinely collected  - Easy manual calculation  - No online tool |
| **Footnotes**: Alp= Alkaline phosphatase; BMI= Body mass index; CRP= C-reactive protein; CT= Chemotherapy; LDH= Lactate dehydrogenase; M= Months; NLR= Neutrophil/Lymphocyte ratio; NR= Not reported; NSCLC= Non-small cell lung cancer; OS= Overall survival; PFS= Progression-free survival; PS= Performance status; RT= Radiotherapy | | | | | | | |

| Continued Supplement Table 2: Overview and quality assessment of decision support systems for incurable patients with recurrent metastatic non-small cell lung cancer | | | | | | | |
| --- | --- | --- | --- | --- | --- | --- | --- |
| **Name DSS** | **Development** | **Aim** | **Predictors** | **Output** | **Validation** | **Model performance** | **User friendiness** |
| Keam nomogram(21) | Retrospective: 2002-2011 (South-Korea)  N=306 stage IIIB/IV NSCLC patients with EGFR mutations, receiving gefitinib or erlotinib | Predict PFS (6, 12, 18 m), for targeted therapy | - Bone metastases  - ECOG PS  - Recurrent or first diagnosis  - Response to EGFR-TKI  - TKI 1^st^ / 2^nd^ / later line | PFS | - | Discriminative ability: -  Van Calster level of calibration: Mean  Reilly level of evidence: Level 1 | - Routinely collected  - Easy manual calculation  - No online tool |
| Kim prognostic score(22) | Retrospective: 2006-2008 (South-Korea)  N=257 stage IIIB/IV NSCLC patients receiving erlotinib | Predict OS and PFS, for targeted therapy | OS:  - ECOG PS  - LDH  - Skin rash  PFS:  - ≥2 CT cycles  - Intra-abdominal  metastases  - Skin rash | 4 prognostic groups based on number of risk factors:  OS  0) Good 🡪 22.0 m  1) Moderate 🡪 9.3 m  2) Poor 🡪 5.4 m  3) Very poor 🡪 2.7 m  PFS  0) Good 🡪 6.5 m  1) Moderate 🡪 3.0 m  2) Poor 🡪 1.2 m  3) Very poor 🡪 0.9 m | - | Discriminative ability: -  Van Calster level of calibration: NR  Reilly level of evidence: Level 1 | - Routinely collected  - Easy manual calculation  - No online tool |
| Clinical modes EGFR-TKI failing(23) | Clinical trial: 2002-2011 (China)  N=120 stage III/IV NSCLC patients with failing EGFR-TKI and N=107 validation cohort | Predict OS and PFS, for targeted therapy | - Development tumor burden  - Duration disease control  - Symptom burden | 3 groups based on disease progression:  OS  1) Dramatic 🡪 17.1 m  2) Gradual 🡪 39.4 m  3) Local 🡪 23.1 m  PFS  1) Dramatic 🡪 9.3 m  2) Gradual 🡪 12.9 m  3) Local 🡪 9.2 m | - | Discriminative ability: -  Van Calster level of calibration: NR  Reilly level of evidence: Level 1 | - Routinely collected  - Manual calculation not easy, but decision tree available  - No online tool |
| Lei score for MSCC(24) | Retrospective: 2005-2015 (China)  N=64 NSCLC patients after surgery | Predict OS (6 m), for surgery vs. symptom management | - ECOG PS  - Number of spinal metastases  - Pre-operative ambulatory status  - Time development motor deficits  - Visceral metastases | 3 prognostic groups:  A) Good: 4-5 🡪 95% (more radical surgery)  B) Moderate: 6-7 🡪 47% (decompression and spine stabilization)  C) Poor: 8-10 🡪 11% (RT and supportive care) | - | Discriminative ability: -  Van Calster level of calibration: NR  Reilly level of evidence: Level 1 | - Routinely collected  - Easy manual calculation  - No online tool |
| **Footnotes**: BMI= Body mass index; CT= Chemotherapy; EGFR-TKI= Epidermal growth factor receptor tyrosine kinase inhibitors; LDH= Lactate dehydrogenase; M= Months; NLR= Neutrophil/Lymphocyte ratio; NR= Not reported; NSCLC= Non-small cell lung cancer; OS= Overall survival; PFS= Progression-free survival; PS= Performance status; RT= Radiotherapy | | | | | | | |

| Continued Supplement Table 2: Overview and quality assessment of decision support systems for incurable patients with recurrent metastatic non-small cell lung cancer | | | | | | | |
| --- | --- | --- | --- | --- | --- | --- | --- |
| **Name DSS** | **Development** | **Aim** | **Predictors** | **Output** | **Validation** | **Model performance** | **User friendiness** |
| Lin prognostic index(25) | Retrospective: 1998-2007 (US)  N=5054 NSCLC (N=625 stage IIIB; N=1533 stage IV), randomly divided into development and validation populations (50%), receiving surgery and/or CT | Predict OS (1-5 years), for mixed treatments | - Age  - Cerebrovascular disease  - CT  - Histology  - Peripheral vascular disease  - Sex  - Smoking  - Stage  - Surgery  - Type 2 diabetes mellitus | 6 risk groups:  1) 0-9  2) 10-14  3) 15-19  4) 20-24  5) 25-29  6) >29 | - | Discriminative ability: Internal(25)  1-year AUC=0.841  2-year AUC=0.849  3-year AUC=0.848  5-year AUC=0.838  Van Calster level of calibration: Mean  Reilly level of evidence: Level 1 | - Routinely collected  - Easy manual calculation  - No online tool |
| Mou prognostic scores(26) | Retrospective: 2008-2013 (China)  N= 227 lung adenocarcinoma (N=212 stage IIIB/IV) receiving 1^st^ line cisplatin or pemetrexed | Predict PFS, for systemic therapy | 0 metastases:  - Age  - Albumin  - CYFRA21-1  - Sex  - Total proteins  - Triglycerides  - Urine acid  1 metastasis:  - Age  - CA199  - Direct bilirubin  - Neuron-specific enolase  2 metastases:  - CA153  - Creatine kinase  - Triglycerides  ≥3 metastases:  - CA125  - CA153  - CA199  - Creatine kinase  - Direct bilirubin  - LDH  - Neuron-specific enolase  - Total bilirubin | 2 risk groups, stratified for number of metastases:  1) Low: < 50^th^ percentile  2) High: > 50^th^ percentile | - | Discriminative ability: -  Van Calster level of calibration: NR  Reilly level of evidence: Level 1 | - Not routinely collected  - Manual calculation not easy  - No online tool |
| **Footnotes**: CA= Cancer antigen; CEA= Carcinoembryonic antigen; CT= Chemotherapy; CYFRA 21-2=Cytokeratin-19 fragments; LDH= Lactate dehydrogenase; M= Months; NLR= Neutrophil/Lymphocyte ratio; NR= Not reported; NSCLC= Non-small cell lung cancer; OS= Overall survival; PFS= Progression-free survival; PS= Performance status; RT= Radiotherapy | | | | | | | |

| Continued Supplement Table 2: Overview and quality assessment of decision support systems for incurable patients with recurrent metastatic non-small cell lung cancer | | | | | | | |
| --- | --- | --- | --- | --- | --- | --- | --- |
| **Name DSS** | **Development** | **Aim** | **Predictors** | **Output** | **Validation** | **Model performance** | **User friendiness** |
| Park prognostic score(27) | Retrospective: 2002-2005 (South-Korea)  N=263 stage IIIB/IV NSCLC patients receiving gefitinib | Predict OS (3-24 m), for targeted therapy | - Albumin  - ALP  - ECOG PS  - Intra-abdominal  metastases  - Progression-free interval prior CT  - Smoking  - Time interval diagnosis to gefitinib  - White blood cell count | 4 prognostic groups based on number of risk factors:  1) Good: 0-1 🡪 18 m  2) Moderate: 2-3 🡪 11.2 m  3) Poor: 4-5 🡪 4 m  4) Very poor: >5 🡪 1.3 m | Retrospective: 2006-2007 (South-Korea)(27)  N=170 NSCLC patients receiving gefitinib | Discriminative ability:  - External(27)  Van Calster level of calibration: NR  Reilly level of evidence: Level 2 | - Not routinely collected  - Manual calculation not easy, but can be derived from table  - Online table(28) |
| Rades prognostic score for MSCC(29) | Retrospective: 1992-2010 (Germany)  N=356 NSCLC with MSCC: N=178 test group and N=178 validation group, receiving short- or long-course RT vs. best supportive care | Predict OS (6 m), for RT vs. symptom management | - ECOG PS  - Pre-RT ambulatory status  - Time until development motor deficits - Visceral metastases | 3 risk groups:  Test  1) High: 6–10 🡪 6%  2) Middle: 11-15 🡪 29%  3) Low: 16-19 🡪 78%  Validation  1) High: 6–10 🡪 4%  2) Middle: 11-15 🡪 24%  3) Low: 16-19 🡪 76% | - | Discriminative ability: -  Van Calster level of calibration: NR  Reilly level of evidence: Level 1 | - Not routinely collected  - Manual calculation not easy, but can be derived from table  - Online calculator tool(30) |
| **DSS for BM: A t/m O**  A) Recursive partitioning analysis (RPA)(31) | RCT: 1979-1993 (US and Canada)  N=1200 patients with BM (N=732 lung cancer), receiving fractionated RT and radiation sensitizers | Predict OS, for RT | - Age  - ECM  - KPS  - Primary tumor under control | 3 risk groups:  - Class I: KPS ≥70, no ECM, age <65, controlled primary tumor 🡪 7.1 m  - Class II: Others 🡪 4.2 m  - Class III: KPS<70 🡪 2.3 m | Retrospective: ? (Norway)(32)  2008-2009 (China)(33)  2006-2010 (China)(34)  2002-2011 (Canada and Netherlands)(35)  N=183 NSCLC with BM, with WBRT +/- SRS or surgery(32)  N=290 NSCLC with BM with WBRT, CT, EGFR-TKI(33)  N=210 NSCLC with BM with WBRT, SRS, surgery, CT and/or EGFR-TKI(34)  N=501 patients with BM (N=286 lung cancer) with SRS (N=381) vs. Fractionated SRT (N=120)(35) | Discriminative ability: External(32-36)  AUC=0.64-0.66 (35)  AUC=0.553 (36)  Van Calster level of calibration: NR  Reilly level of evidence: Level 3 | - Routinely collected  - Easy manual calculation  - Online calculator tool(37) |
| **Footnotes**: Alp= Alkaline phosphatase; AUC= Area under the ROC curve; BM= Brain metastases; CT= Chemotherapy; ECM= Extracranial metastases; EGFR-TKI= Epidermal growth factor receptor tyrosine kinase inhibitors; KPS= Karnofsky Performance status; M= Months; MSCC= Metastatic spinal cord compression; NR= Not reported; NSCLC= Non-small cell lung cancer; OS= Overall survival; PS= Performance status; RCT= Randomized clinical trial; RT= Radiotherapy; SRS= Stereotactic radiosurgery; SRT= Stereotactic radiotherapy; WBRT= Whole brain radiotherapy | | | | | | | |

| Continued Supplement Table 2: Overview and quality assessment of decision support systems for incurable patients with recurrent metastatic non-small cell lung cancer | | | | | | | | |
| --- | --- | --- | --- | --- | --- | --- | --- | --- |
| **Name DSS** | **Development** | | **Aim** | **Predictors** | **Output** | **Validation** | **Model performance** | **User friendiness** |
| **DSS for BM: A t/m O continued**  B) Rotterdam score (RDAM)(38) | Retrospective: 1981-1990 (Netherlands)  N=1292 (N=721 lung cancer) patients with BM, receiving WBRT, SRS, SRT and/or surgery | | Predict OS, for RT | - KPS  - Controlled primary tumor  - Response to steroids | 3 prognostic groups:  1) Good: ECOG PS 0-1; good response steroids 🡪 6.3 m  2) Moderate: Others 🡪 3.4 m  3) Poor: ECOG PS 2-3; little response steroids; limited tumor control 🡪 1.3 m | Retrospective: year? (Norway)(32)  2002-2011 (Canada and Netherlands) (35)  N=183 NSCLC patients with BM, with WBRT +/-SRS or surgery(32)  N=501 BM (N=286 lung cancer) with SRS (N=381) vs. Fractionated SRT (N=120) (35) | Discriminative ability:  External(32, 35)  AUC=0.60-0.63 (35)  Van Calster level of calibration: NR  Reilly level of evidence: Level 3 | - Routinely collected  - Easy manual calculation  - No online tool |
| C) Score index for radiosurgery (SIR)(39) | | Retrospective: 1993-1997 (Brazil)  N=65 patients with BM (N=30 NSCLC), receiving SRS  WBRT, SRT and/or surgery | Predict OS, for RT | - Age  - Controlled primary tumor  - KPS  - Lesion volume | 3 prognostic groups:  1) Good: 8-10 🡪 31.4 m  2) Moderate: 4-7 🡪 7 m  3) Poor: 1-3 🡪 2.9 m | Retrospective: year? (Norway)(32)  2002-2011 (Canada and Netherlands) (35)  N=183 NSCLC with BM, with WBRT +/- SRS or surgery(32)  N=501 BM (N=286 lung cancer) with SRS (N=381) vs. Fractionated SRT (N=120) (35) | Discriminative ability:  External(32, 35)  AUC=0.55-0.58 (35)  Van Calster level of calibration: NR  Reilly level of evidence: Level 3 | - Routinely collected  - Easy manual calculation  - No online tool |
| D) Modified RPA I (mRPA)(40) | | Retrospective: 1985-2000 (Germany)  N=916 patients with BM (N=424 lung cancer), receiving SRS, WBRT, SRT and/or surgery | Predict OS, for RT | - Controlled primary tumor  - ECM  - KPS  - Number of BM | 3 risk groups:  - Class I: KPS ≥70, no ECM, age <65, controlled primary tumor, single BM 🡪 8.2 m  - Class II: Others 🡪 4.9 m  - Class IIIa: KPS<70, no ECM, age <65, controlled primary tumor, single BM 🡪 3.2 m  - Class IIIb: KPS<70, others from Class III 🡪 1.9 m  - Class IIIc: KPS<70, ECM, age ≥65, uncontrolled primary tumor, multiple BM🡪 1.2 m | - | Discriminative ability: -  Van Calster level of calibration: NR  Reilly level of evidence: Level 1 | - Routinely collected  - Easy manual calculation  - No online tool |
| **Footnotes**: AUC= Area under the ROC curve; BM= Brain metastases; ECM= Extracranial metastases; KPS= Karnofsky Performance status; M= Months; NR= Not reported; NSCLC= Non-small cell lung cancer; OS= Overall survival; PFS= Progression-free survival; PS= Performance status; RT= Radiotherapy; SRS= Stereotactic radiosurgery; SRT= Stereotactic radiotherapy; WBRT= Whole brain radiotherapy | | | | | | | | |

| Continued Supplement Table 2: Overview and quality assessment of decision support systems for incurable patients with recurrent metastatic non-small cell lung cancer | | | | | | | |
| --- | --- | --- | --- | --- | --- | --- | --- |
| **Name DSS** | **Development** | **Aim** | **Predictors** | **Output** | **Validation** | **Model performance** | **User friendiness** |
| **DSS for BM: A t/m O continued**  E) Basic score for brain metastases (BSBM)(41) | Prospective: 1999-2003 (Belgium)  N=113 patients with BM (N=57 lung cancer), receiving GKRS | Predict OS, for RT | - Controlled primary tumor  - ECM  - KPS | 4 prognostic groups:  0) Very poor 🡪1.9 m  1) Poor 🡪 3.3 m  2) Moderate 🡪 13.1 m  3) Good 🡪 55% alive at 32 m | Retrospective: year? (Norway)(32)  2002-2011 (Canada and Netherlands) (35)  N=183 NSCLC with BM, with WBRT +/- SRS or surgery(32)  N=501 BM (N=286 lung cancer) with SRS (N=381) vs. Fractionated SRT (N=120) (35) | Discriminative ability:  External(32, 35)  AUC=0.62-0.67 (35)  Van Calster level of calibration: NR  Reilly level of evidence: Level 3 | - Routinely collected  - Easy manual calculation  - No online tool |
| F) Graded prognostic assessment (GPA)(42) | Clinical trials: year? (US and Canada)  N=1960 patients with BM, receiving WBRT, SRS, SRT and/or surgery | Predict OS, for RT | - Age  - ECM  - KPS  - Number of BM | 4 prognostic groups:  3.5-4) Good 🡪 11 m  3) Moderate 🡪 6.9 m  1.5-2.5) Poor 🡪 3.8 m  0-1) Very poor 🡪 2.6 m | Retrospective: year? (Norway) (32)  2008-2009 (China) (33)  1982-2004 (US)(43)  2006-2010 (China) (34)  2002-2011 (Canada and Netherlands) (35)  1996-2001 (US)(44)  N=183 NSCLC with BM, with WBRT +/- SRS or surgery(32)  N=290 NSCLC with BM, with WBRT, CT, EGFR-TKI(33)  N=780 NSCLC with BM(43)  N=210 NSCLC with BM, with WBRT vs. WBRT and SRS or surgery +/- CT or EGFR-TKI(34)  N=501 BM (N=286 lung cancer), with SRS (N=381) vs. Fractionated SRT (N=120) (35)  N=252 BM (N=211 lung cancer), with WBRT +/- SRS(44) | Discriminative ability:  External(32) (33, 34) (35, 43, 44)  AUC=0.58-0.59 (35)  Van Calster level of calibration: NR  Reilly level of evidence: Level 3 | - Routinely collected  - Easy manual calculation  - Online calculator tool(45) |
| G) Golden Grading System (GGS)(46) | Retrospective: 1991-2005 (US)  N=479 patients with BM (N=169 lung cancer), receiving GKRS or SRS with/without WBRT | Predict OS, for RT | - Age  - ECM  - KPS | 4 prognostic groups:  0) Good 🡪 20.6 m  1) Moderate 🡪 16.9 m  2) Poor 🡪 9.1 m  3) Very poor 🡪 6.6 m | Retrospective: year? (Norway) (32)  2002-2011 (Canada and Netherlands) (35)  N=183 NSCLC with BM, with WBRT +/- SRS or surgery(32)  N=501 BM (N=286 lung cancer), with SRS (N=381) vs. Fractionated SRT (N=120) (35) | Discriminative ability:  External(32, 35)  AUC=0.64-0.69 (35)  Van Calster level of calibration: NR  Reilly level of evidence: Level 3 | - Routinely collected  - Easy manual calculation  - No online tool |
| **Footnotes**: AUC= Area under the ROC curve; BM= Brain metastases; ECM= Extracranial metastases; GKRS= Gamma knife radiosurgery; KPS= Karnofsky Performance status; M= Months; NR= Not reported; NSCLC= Non-small cell lung cancer; OS= Overall survival; PFS= Progression-free survival; PS= Performance status; RT= Radiotherapy; SRS= Stereotactic radiosurgery; SRT= Stereotactic radiotherapy; WBRT= Whole brain radiotherapy | | | | | | | |

| Continued Supplement Table 2: Overview and quality assessment of decision support systems for incurable patients with recurrent metastatic non-small cell lung cancer | | | | | | | |
| --- | --- | --- | --- | --- | --- | --- | --- |
| **Name DSS** | **Development** | **Aim** | **Predictors** | **Output** | **Validation** | **Model performance** | **User friendiness** |
| **DSS for BM: A t/m O continued**  H) RADES I(47) | Retrospective: 1992-2005 (Germany)  N=1085 patients with BM (N=525 lung cancer), receiving WBRT | Predict OS, for RT | - Age  - ECM  - KPS  - Time interval diagnosis until RT | 4 prognostic groups:  6-mnth OS  A) Very poor (9-10) 🡪 6%  B) Poor (11-13) 🡪 15%  C) Moderate (14-16) 🡪 43%  D) Good (17-18) 🡪 76%  12-mnth OS  A) Very poor (9-10) 🡪 1%  B) Poor (11-13) 🡪 7%  C) Moderate (14-16) 🡪 22%  D) Good (17-18) 🡪 49% | Retrospective: year? (Norway) (32)  2002-2011 (Canada and Netherlands) (35)  N=183 NSCLC patients with BM, receiving WBRT +/- SRS or surgery(32)  N=501 patients with BM (N=286 lung cancer), receiving SRS (N=381) vs. Fractionated SRT (N=120)(35) | Discriminative ability:  External(32, 35)  AUC=0.65-0.69 (35)  Van Calster level of calibration: NR  Reilly level of evidence: Level 3 | - Routinely collected  - Easy manual calculation  - No online tool |
| I) Disease-specific GPA (DS-GPA)(48) | Retrospective: 1985-2007 (US)  N=4259 patients with BM (N=1888 NSCLC), receiving WBRT, SRS, SRT and/or surgery | Predict OS, for RT | - Age  - ECM  - EGFR  - KPS  - Number of BM | 4 prognostic groups:  3.5-4) Good 🡪 14.8 m  3) Moderate 🡪 11.3 m  1.5-2.5) Poor 🡪 6.5 m  0-1) Very poor 🡪 3.0 m | Retrospective: 2002-2011 (Canada and Netherlands) (35)  1998-2011 (Japan)(49)  2007-2011 (South-Korea)(50)  1996-2001 (US)(44)  N=501 patients with BM (N=286 lung cancer), receiving SRS (N=381) vs. Fractionated SRT (N=120)(35)  N=4608 patients with BM (N=2827 NSCLC), receiving GKRS(49)  N=292 lung adenocarcinoma patients with BM(50)  N=252 patients with BM (N=211 lung cancer), receiving WBRT +/- SRS(44) | Discriminative ability:  External(35, 36, 49, 50)  AUC=0.61-0.64 (35, 44)  AUC=0.579 (36)  Van Calster level of calibration: NR  Reilly level of evidence: Level 3 | - Routinely collected  - Easy manual calculation  - Online calculator tool(45) |
| J) RADES II(51) | Retrospective: year? (Germany)  N=1797 patients with BM: N=1198 in test cohort and N=599 in validation cohort, receiving WBRT, SRS, SRT and/or surgery | Predict OS, for RT | - Age  - ECM  - KPS  - Number of BM  - Time interval diagnosis until RT | 3 prognostic groups:  6-m OS test:  A) Poor (14-18) 🡪 9%  B) Moderate (19-23) 🡪 41%  C) Good (24-27) 🡪 78%  6-m OS validation:  A) Poor (14-18) 🡪 7%  B) Moderate (19-23) 🡪 39%  C) Good (24-27) 🡪 79% | Retrospective: year? (Norway) (32)  2002-2011 (Canada and Netherlands) (35)  N=183 patients with NSCLC with BM, receiving WBRT +/- SRS or surgery(32)  N=501 patients with BM (N=286 lung cancer), receiving SRS (N=381) vs. Fractionated SRT (N=120) (35) | Discriminative ability:  External(32, 35)  AUC=0.60-0.64 (35)  Van Calster level of calibration: NR  Reilly level of evidence: Level 3 | - Routinely collected  - Easy manual calculation  - No online tool |
| **Footnotes**: AUC= Area under the ROC curve; BM= Brain metastases; ECM= Extracranial metastases; GKRS= Gamma knife radiosurgery; KPS= Karnofsky Performance status; M= Months; NR= Not reported; NSCLC= Non-small cell lung cancer; OS= Overall survival; PFS= Progression-free survival; PS= Performance status; RT= Radiotherapy; SRS= Stereotactic radiosurgery; SRT= Stereotactic radiotherapy; WBRT= Whole brain radiotherapy | | | | | | | |

| Continued Supplement Table 2: Overview and quality assessment of decision support systems for incurable patients with recurrent metastatic non-small cell lung cancer | | | | | | | |
| --- | --- | --- | --- | --- | --- | --- | --- |
| **Name DSS** | **Development** | **Aim** | **Predictors** | **Output** | **Validation** | **Model performance** | **User friendiness** |
| **DSS for BM: A t/m O continued**  K) Barnholtz-Sloan (BS) nomogram(36) | Clinical trials: year? (US)  N=2367 patients with BM (N=1555 lung cancer), receiving WBRT, SRS, SRT and/or surgery | Predict OS, for RT | - Age  - Controlled primary tumor  - ECM  - Histology  - KPS  - Number of BM | Nomogram | - | Discriminative ability:  Internal(36)  AUC=0.604  Van Calster level of calibration: Moderate  Reilly level of evidence: Level 1 | - Routinely collected  - Easy manual calculation  - Nomogram and online calculator tool(52) |
| L) mRPA II (49) | Retrospective: 1998-2008 (Japan)  N=3753 patients with BM: N=2000 test cohort (N=1283 lung cancer) and N=1753 validation cohort (N=1183 lung cancer), receiving WBRT, SRS, SRT and/or surgery | Predict OS, for RT | - Controlled primary tumor  - ECM  - KPS  - Number of BM | 5 risk groups:  - Class I: KPS ≥70, no ECM, age <65, primary tumor controlled, single BM 🡪 20.4 m  - Class IIa: 0-1 risk factors 🡪 15.8 m  - Class IIb: 2 risk factors 🡪 9.8 m  - Class IIc: 3-4 risk factors 🡪4.7 m  - Class III: KPS<70 🡪 2.2 m | - | Discriminative ability:  Internal(49)  Van Calster level of calibration: NR  Reilly level of evidence: Level 1 | - Routinely collected  - Easy manual calculation  - No online tool |
| M) NSCLC-specific RADES (NSCLC-RADES)(53) | Retrospective: year? (Germany)  N=514 NSCLC patients with BM: N=257 test cohort and N=257 validation cohort, receiving WBRT | Predict OS, for RT | - ECM  - KPS  - Sex | 3 prognostic groups:  6-m OS test:  A) Poor (5-9) 🡪 9%  B) Moderate (11-12) 🡪 54%  C) Good (15) 🡪 79%  6-m OS validation:  A) Poor (5-9) 🡪 14%  B) Moderate (11-12) 🡪 56%  C) Good (15) 🡪 78% | - | Discriminative ability: -  Van Calster level of calibration: NR  Reilly level of evidence: Level 1 | - Routinely collected  - Easy manual calculation  - No online tool |
| O) Modified BSBM (mBSBM)(54) | Retrospective: 1998-2013 (Japan)  N=2838 patients with BM (N=1868 lung cancer, N=1604 NSCLC), receiving SRS GKRS or WBRT | Predict OS and neurological OS (1 year), for RT | - Controlled primary tumor  - ECM  - KPS  - Lesion volume  - Meningeal  dissemination  - Neurological  symptoms  - Number of BM | 4 prognostic groups with 2 subclasses based on neurological symptoms (A good vs. B poor):  1-year OS:  0) Very poor 🡪 A) 64.6% vs. B) 45%  1) Poor 🡪 A) 82.5% vs. B) 63.3%  2) Moderate 🡪 A) 86.4% vs. B) 73.7%  3) Good 🡪 A) 91.4% vs. B) 73.5%  1-year neurological OS:  0) Very poor 🡪 A) 82.6% vs. B) 52.4%  1) Poor 🡪 A) 90.5% vs. B) 78.1%  2) Moderate 🡪 A) 91.1% vs. B) 83.2%  3) Good 🡪 A) 93.9% vs. B) 76.3% | - | Discriminative ability: -  Van Calster level of calibration: NR  Reilly level of evidence: Level 1 | - Routinely collected  - Easy manual calculation  - No online tool |
| **Footnotes**: AUC= Area under the ROC curve; BM= Brain metastases; ECM= Extracranial metastases; GKRS= Gamma knife radiosurgery; KPS= Karnofsky Performance status; M= Months; NR= Not reported; NSCLC= Non-small cell lung cancer; OS= Overall survival; PFS= Progression-free survival; PS= Performance status; RT= Radiotherapy; SRS= Stereotactic radiosurgery; SRT= Stereotactic radiotherapy; WBRT= Whole brain radiotherapy | | | | | | | |

| Continued Supplement Table 2: Overview and quality assessment of decision support systems for incurable patients with recurrent metastatic non-small cell lung cancer | | | | | | | |
| --- | --- | --- | --- | --- | --- | --- | --- |
| **Name DSS** | **Development** | **Aim** | **Predictors** | **Output** | **Validation** | **Model performance** | **User friendiness** |
| Sanchez-Lara prognostic score(55) | Prospective: 2009-2011 (Mexico)  N=119 stage IIIB/IV NSCLC patients receiving 1^st^ line CT: paclitaxel and cisplatin | Predict OS and HRQL (1 year), for systemic therapy | - ECOG PS  - Phase angle  - Subjective global assessment | 3 risk groups based on regression coefficients:  1) Low (0-1.9) 🡪 78.4%  2) Moderate (2-3) 🡪 53%  3) High (>3.1) 🡪 13.8% | - | Discriminative ability: -  Van Calster level of calibration: NR  Reilly level of evidence: Level 1 | - Not routinely collected  - Manual calculation not easy  - No online tool |
| Revised Tokuhashi score(56) | Retrospective + prospective: ~1998 (Japan)  N=246 patients with spinal metastases deceased: N=164 after tumor excision and N= 82 after palliative surgery; N=118 patients prospectively followed after 1998 with impact analysis of score, receiving surgery vs. conservative treatment vs. palliative treatment | Predict OS, for surgery vs. symptom management | - General condition  - Metastases major internal organs  - Number of extraspinal bone metastases  - Number of spinal metastases  - Primary tumor location  - Severity palsy | 3 prognostic groups:  1) Good (12-15) 🡪 ≥12 m in 87.5%  2) Moderate (9-11) 🡪 6-12 m in 78.6%  3) Poor (0-8) 🡪 <6 m in 89% | Prospective impact analysis: 1987-? (Japan)(57)  Retrospective: 2008-2013 (China)(58)  N=183 spinal metastases (N=46 lung cancer) followed after application of revised score(57)  N=151 lung cancer patients with spinal metastases(58) | Discriminative ability:  External(57, 58)  - Consistency predicted vs. observed prognosis impact analysis: 87.9% (57)  - Predicted vs. Observed survival in 8.2% (58)  Van Calster level of calibration: Mean  Reilly level of evidence: Level 4 | - Routinely collected  - Easy manual calculation and decision tree available  - Online calculator tool(59) |
| Zhang prognostic score (60) | Retrospective: 1998-2011 (US)  N=1161 stage IIIB/IV NSCLC patients: N=773 test cohort and N=388 validation cohort, receiving surgery, CT, RT | Predict OS (1 year), for mixed treatments | - Albumin  - ALP  - International normalised ratio - Proteins  - Urea nitrogen | 3 risk groups based on tertiles:  1-year OS test:  1) Low 🡪 16.9 m  2) Moderate 🡪 7.2 m  3) High 🡪 2.1 m  1-year OS validation:  1) Low 🡪 15.1 m  2) Moderate 🡪 7.6 m  3) High 🡪 2.6 m | - | Discriminative ability:  Internal (60)  Test  AUC=0.79  Validation  AUC=0.83  Van Calster level of calibration: NR  Reilly level of evidence: Level 1 | - Routinely collected  - Manual calculation not easy  - No online tool |
| **Footnotes**: ALP= Alkaline Phosphatase; AUC= Area under the ROC curve; HRQL= Health-related quality of life; M= Months; NR= Not reported; NSCLC= Non-small cell lung cancer; OS= Overall survival; PFS= Progression-free survival; PS= Performance status | | | | | | | |

**References**

1. Trape J, Montesinos J, Catot S, Buxo J, Franquesa J, Sala M, et al. A prognostic score based on clinical factors and biomarkers for advanced non-small cell lung cancer. The International journal of biological markers. 2012;27(3):e257-62.

2. Armero C, Cabras S, Castellanos ME, Perra S, Quiros A, Oruezabal MJ, et al. Bayesian analysis of a disability model for lung cancer survival. Statistical methods in medical research. 2016;25(1):336-51.

3. Blanchon F, Grivaux M, Asselain B, Lebas FX, Orlando JP, Piquet J, et al. 4-year mortality in patients with non-small-cell lung cancer: development and validation of a prognostic index. Lancet Oncol. 2006;7(10):829-36.

4. Wang X, Gu L, Zhang Y, Sargent DJ, Richards W, Ganti AK, et al. Validation of survival prognostic models for non-small-cell lung cancer in stage- and age-specific groups. Lung cancer (Amsterdam, Netherlands). 2015;90(2):281-7.

5. Daniele S, Sandro B, Salvatore I, Alfredo F, Francesco F, Domenico G, et al. Natural History of Non-Small-Cell Lung Cancer with Bone Metastases. Scientific reports. 2015;5:18670.

6. Di Maio M, Lama N, Morabito A, Smit EF, Georgoulias V, Takeda K, et al. Clinical assessment of patients with advanced non-small-cell lung cancer eligible for second-line chemotherapy: a prognostic score from individual data of nine randomised trials. European journal of cancer (Oxford, England : 1990). 2010;46(4):735-43.

7. Di Maio M, Krzakowski M, Fougeray R, Kowalski DM, Gridelli C. Prognostic score for second-line chemotherapy of advanced non-small-cell lung cancer: external validation in a phase III trial comparing vinflunine with docetaxel. Lung cancer (Amsterdam, Netherlands). 2012;77(1):116-20.

8. Florescu M, Hasan B, Seymour L, Ding K, Shepherd FA. A clinical prognostic index for patients treated with erlotinib in National Cancer Institute of Canada Clinical Trials Group study BR.21. Journal of thoracic oncology : official publication of the International Association for the Study of Lung Cancer. 2008;3(6):590-8.

9. Wang F, Zhang Y, Zhao H, Chen L, Shi YX, Zhang L. Validation of a clinical prognostic model in Chinese patients with metastatic and advanced pretreated non-small cell lung cancer treated with gefitinib. Medical oncology (Northwood, London, England). 2011;28(1):331-5.

10. Wojas-Krawczyk K, Krawczyk P, Mlak R, Kucharczyk T, Kowalski DM, Krzakowski M, et al. The applicability of a predictive index for second- and third-line treatment of unselected non-small-cell lung cancer patients. Respiration; international review of thoracic diseases. 2011;82(4):341-50.

11. Forrest LM, McMillan DC, McArdle CS, Angerson WJ, Dunlop DJ. Evaluation of cumulative prognostic scores based on the systemic inflammatory response in patients with inoperable non-small-cell lung cancer. British journal of cancer. 2003;89(6):1028-30.

12. Jiang AG, Chen HL, Lu HY. Comparison of Glasgow prognostic score and prognostic index in patients with advanced non-small cell lung cancer. Journal of cancer research and clinical oncology. 2015;141(3):563-8.

13. Fan H, Shao ZY, Xiao YY, Xie ZH, Chen W, Xie H, et al. Comparison of the Glasgow Prognostic Score (GPS) and the modified Glasgow Prognostic Score (mGPS) in evaluating the prognosis of patients with operable and inoperable non-small cell lung cancer. Journal of cancer research and clinical oncology. 2016.

14. Prognostic Scores of Forrest et al for a Patient with Inoperable Non-Small-Cell Carcinoma of the Lung (Glasgow Prognostic Score) [Available from: <http://www.medicalalgorithms.com/prognostic-scores-of-forrest-et-al-for-a-patient-with-inoperable-non-small-cell-carcinoma-of-the-lung-glasgow-prognostic-score>.

15. McMillan DC, Crozier JE, Canna K, Angerson WJ, McArdle CS. Evaluation of an inflammation-based prognostic score (GPS) in patients undergoing resection for colon and rectal cancer. International journal of colorectal disease. 2007;22(8):881-6.

16. Kasymjanova G, MacDonald N, Agulnik JS, Cohen V, Pepe C, Kreisman H, et al. The predictive value of pre-treatment inflammatory markers in advanced non-small-cell lung cancer. Curr Oncol. 2010;17(4):52-8.

17. Jafri SH, Shi R, Mills G. Advance lung cancer inflammation index (ALI) at diagnosis is a prognostic marker in patients with metastatic non-small cell lung cancer (NSCLC): a retrospective review. BMC Cancer. 2013;13:158.

18. Gagnon B, Agulnik JS, Gioulbasanis I, Kasymjanova G, Morris D, MacDonald N. Montreal prognostic score: estimating survival of patients with non-small cell lung cancer using clinical biomarkers. British journal of cancer. 2013;109(8):2066-71.

19. Ulas A, Turkoz FP, Silay K, Tokluoglu S, Avci N, Oksuzoglu B, et al. A laboratory prognostic index model for patients with advanced non-small cell lung cancer. PLoS One. 2014;9(12):e114471.

20. Hoang T, Dahlberg SE, Sandler AB, Brahmer JR, Schiller JH, Johnson DH. Prognostic models to predict survival in non-small-cell lung cancer patients treated with first-line paclitaxel and carboplatin with or without bevacizumab. Journal of thoracic oncology : official publication of the International Association for the Study of Lung Cancer. 2012;7(9):1361-8.

21. Keam B, Kim DW, Park JH, Lee JO, Kim TM, Lee SH, et al. Nomogram Predicting Clinical Outcomes in Non-small Cell Lung Cancer Patients Treated with Epidermal Growth Factor Receptor Tyrosine Kinase Inhibitors. Cancer research and treatment : official journal of Korean Cancer Association. 2014;46(4):323-30.

22. Kim ST, Lee J, Sun JM, Park YH, Ahn JS, Park K, et al. Prognostic model to predict outcomes in non-small cell lung cancer patients with erlotinib as salvage treatment. Oncology. 2010;79(1-2):78-84.

23. Yang JJ, Chen HJ, Yan HH, Zhang XC, Zhou Q, Su J, et al. Clinical modes of EGFR tyrosine kinase inhibitor failure and subsequent management in advanced non-small cell lung cancer. Lung cancer (Amsterdam, Netherlands). 2013;79(1):33-9.

24. Lei M, Liu Y, Tang C, Yang S, Liu S, Zhou S. Prediction of survival prognosis after surgery in patients with symptomatic metastatic spinal cord compression from non-small cell lung cancer. BMC Cancer. 2015;15:853.

25. Lin J, Carter CA, McGlynn KA, Zahm SH, Nations JA, Anderson WF, et al. A Prognostic Model to Predict Mortality among Non-Small-Cell Lung Cancer Patients in the U.S. Military Health System. Journal of thoracic oncology : official publication of the International Association for the Study of Lung Cancer. 2015;10(12):1694-702.

26. Mou W, Liu Z, Luo Y, Zou M, Ren C, Zhang C, et al. Development and cross-validation of prognostic models to assess the treatment effect of cisplatin/pemetrexed chemotherapy in lung adenocarcinoma patients. Medical Oncology. 2014;31(9):1-9.

27. Park MJ, Lee J, Hong JY, Choi MK, Yi JH, Lee SJ, et al. Prognostic model to predict outcomes in nonsmall cell lung cancer patients treated with gefitinib as a salvage treatment. Cancer. 2009;115(7):1518-30.

28. Model of Park et al. for Predicting Outcome for a Patient with a Nonsmall Cell Lung Cancer Treated with Gefitinib As Salvage Therapy [Available from: <http://www.medicalalgorithms.com/model-of-park-et-al-for-predicting-outcome-for-a-patient-with-a-nonsmall-cell-lung-cancer-treated-with-gefitinib-as-salvage-therapy>.

29. Rades D, Douglas S, Veninga T, Schild SE. A validated survival score for patients with metastatic spinal cord compression from non-small cell lung cancer. BMC Cancer. 2012;12:302.

30. Survival Model for Patient Undergoing Radiotherapy for Metastatic Spinal Cord Compression (Rades) [Available from: <http://www.medicalalgorithms.com/metastatic-spinal-cord-compression>.

31. Gaspar L, Scott C, Rotman M, Asbell S, Phillips T, Wasserman T, et al. Recursive partitioning analysis (RPA) of prognostic factors in three Radiation Therapy Oncology Group (RTOG) brain metastases trials. Int J Radiat Oncol Biol Phys. 1997;37(4):745-51.

32. Nieder C, Bremnes RM, Andratschke NH. Prognostic scores in patients with brain metastases from non-small cell lung cancer. Journal of thoracic oncology : official publication of the International Association for the Study of Lung Cancer. 2009;4(11):1337-41.

33. Sun CX, Li T, Zheng X, Cai JF, Meng XL, Yang HJ, et al. Recursive partitioning analysis classification and graded prognostic assessment for non-small cell lung cancer patients with brain metastasis: a retrospective cohort study. Chinese journal of cancer research = Chung-kuo yen cheng yen chiu. 2011;23(3):177-82.

34. Fan Y, Huang Z, Fang L, Miu L, Lin N, Gong L, et al. Chemotherapy and EGFR tyrosine kinase inhibitors for treatment of brain metastases from non-small-cell lung cancer: survival analysis in 210 patients. OncoTargets and therapy. 2013;6:1789-803.

35. Rodrigues G, Gonzalez-Maldonado S, Bauman G, Senan S, Lagerwaard F. A statistical comparison of prognostic index systems for brain metastases after stereotactic radiosurgery or fractionated stereotactic radiation therapy. Clin Oncol (R Coll Radiol). 2013;25(4):227-35.

36. Barnholtz-Sloan JS, Yu C, Sloan AE, Vengoechea J, Wang M, Dignam JJ, et al. A nomogram for individualized estimation of survival among patients with brain metastasis. Neuro Oncol. 2012;14(7):910-8.

37. RTOG Recursive Partitioning Analysis (RPA) of Gaspar et al for Patients with Brain Metastases [Available from: [www.medicalalgorithms.com/rtog-recursive-partitioning-analysis-rpa-of-gaspar-et-al-for-patients-with-brain-metastases](http://www.medicalalgorithms.com/rtog-recursive-partitioning-analysis-rpa-of-gaspar-et-al-for-patients-with-brain-metastases).

38. Lagerwaard FJ, Levendag PC, Nowak PJ, Eijkenboom WM, Hanssens PE, Schmitz PI. Identification of prognostic factors in patients with brain metastases: a review of 1292 patients. Int J Radiat Oncol Biol Phys. 1999;43(4):795-803.

39. Weltman E, Salvajoli JV, Brandt RA, de Morais Hanriot R, Prisco FE, Cruz JC, et al. Radiosurgery for brain metastases: a score index for predicting prognosis. Int J Radiat Oncol Biol Phys. 2000;46(5):1155-61.

40. Lutterbach J, Bartelt S, Stancu E, Guttenberger R. Patients with brain metastases: hope for recursive partitioning analysis (RPA) class 3. Radiotherapy and oncology : journal of the European Society for Therapeutic Radiology and Oncology. 2002;63(3):339-45.

41. Lorenzoni J, Devriendt D, Massager N, David P, Ruiz S, Vanderlinden B, et al. Radiosurgery for treatment of brain metastases: estimation of patient eligibility using three stratification systems. Int J Radiat Oncol Biol Phys. 2004;60(1):218-24.

42. Sperduto PW, Berkey B, Gaspar LE, Mehta M, Curran W. A new prognostic index and comparison to three other indices for patients with brain metastases: an analysis of 1,960 patients in the RTOG database. Int J Radiat Oncol Biol Phys. 2008;70(2):510-4.

43. Guo S, Reddy CA, Chao ST, Suh JH, Barnett GH, Vogelbaum MA, et al. Impact of non-small cell lung cancer histology on survival predicted from the graded prognostic assessment for patients with brain metastases. Lung cancer (Amsterdam, Netherlands). 2012;77(2):389-93.

44. Sperduto PW, Shanley R, Luo X, Andrews D, Werner-Wasik M, Valicenti R, et al. Secondary analysis of RTOG 9508, a phase 3 randomized trial of whole-brain radiation therapy versus WBRT plus stereotactic radiosurgery in patients with 1-3 brain metastases; poststratified by the graded prognostic assessment (GPA). Int J Radiat Oncol Biol Phys. 2014;90(3):526-31.

45. Graded Prognostic Assessment [Available from: <http://brainmetgpa.com/>.

46. Golden DW, Lamborn KR, McDermott MW, Kunwar S, Wara WM, Nakamura JL, et al. Prognostic factors and grading systems for overall survival in patients treated with radiosurgery for brain metastases: variation by primary site. Journal of neurosurgery. 2008;109 Suppl:77-86.

47. Rades D, Dunst J, Schild SE. A new scoring system to predicting the survival of patients treated with whole-brain radiotherapy for brain metastases. Strahlentherapie und Onkologie : Organ der Deutschen Rontgengesellschaft [et al]. 2008;184(5):251-5.

48. Sperduto PW, Chao ST, Sneed PK, Luo X, Suh J, Roberge D, et al. Diagnosis-specific prognostic factors, indexes, and treatment outcomes for patients with newly diagnosed brain metastases: a multi-institutional analysis of 4,259 patients. Int J Radiat Oncol Biol Phys. 2010;77(3):655-61.

49. Yamamoto M, Serizawa T, Sato Y, Kawabe T, Higuchi Y, Nagano O, et al. Validity of two recently-proposed prognostic grading indices for lung, gastro-intestinal, breast and renal cell cancer patients with radiosurgically-treated brain metastases. Journal of neuro-oncology. 2013;111(3):327-35.

50. Lee DW, Shin DY, Kim JW, Keam B, Kim TM, Kim HJ, et al. Additional prognostic role of EGFR activating mutations in lung adenocarcinoma patients with brain metastasis: integrating with lung specific GPA score. Lung cancer (Amsterdam, Netherlands). 2014;86(3):363-8.

51. Rades D, Dziggel L, Haatanen T, Veninga T, Lohynska R, Dunst J, et al. Scoring systems to estimate intracerebral control and survival rates of patients irradiated for brain metastases. Int J Radiat Oncol Biol Phys. 2011;80(4):1122-7.

52. Brain Metastasis nomogram [Available from: <https://apervita.com/community/calculator/individualized-estimation-survival-among-patients-brain-metastasis>.

53. Rades D, Dziggel L, Segedin B, Oblak I, Nagy V, Marita A, et al. A new survival score for patients with brain metastases from non-small cell lung cancer. Strahlentherapie und Onkologie : Organ der Deutschen Rontgengesellschaft [et al]. 2013;189(9):777-81.

54. Serizawa T, Higuchi Y, Nagano O, Matsuda S, Ono J, Saeki N, et al. A new grading system focusing on neurological outcomes for brain metastases treated with stereotactic radiosurgery: the modified Basic Score for Brain Metastases. Journal of neurosurgery. 2014;121 Suppl:35-43.

55. Sanchez-Lara K, Turcott JG, Juarez E, Guevara P, Nunez-Valencia C, Onate-Ocana LF, et al. Association of nutrition parameters including bioelectrical impedance and systemic inflammatory response with quality of life and prognosis in patients with advanced non-small-cell lung cancer: a prospective study. Nutr Cancer. 2012;64(4):526-34.

56. Tokuhashi Y, Matsuzaki H, Oda H, Oshima M, Ryu J. A revised scoring system for preoperative evaluation of metastatic spine tumor prognosis. Spine (Phila Pa 1976). 2005;30(19):2186-91.

57. Tokuhashi Y, Ajiro Y, Umezawa N. Outcome of treatment for spinal metastases using scoring system for preoperative evaluation of prognosis. Spine (Phila Pa 1976). 2009;34(1):69-73.

58. Yu W, Tang L, Lin F, Yao Y, Shen Z. Accuracy of Tokuhashi score system in predicting survival of lung cancer patients with vertebral metastasis. Journal of neuro-oncology. 2015;125(2):427-33.

59. Prognostic Scoring System of Tokuhashi et al for Preoperative Evaluation of a Patient with Metastases to the Spine [Available from: <http://www.medicalalgorithms.com/prognostic-scoring-system-of-tokuhashi-et-al-for-preoperative-evaluation-of-a-patient-with-metastases-to-the-spine>.

60. Zhang K, Lai Y, Axelrod R, Campling B, Hyslop T, Civan J, et al. Modeling the overall survival of patients with advanced-stage non-small cell lung cancer using data of routine laboratory tests. International journal of cancer Journal international du cancer. 2015;136(2):382-91.
